# Anion Exchange Ionomer Binders for Alkaline Fuel Cells

**DOI:** 10.3390/ma18184354

**Published:** 2025-09-17

**Authors:** Alannah C. Gowling, Kelly M. Meek

**Affiliations:** Department of Chemical and Materials Engineering, Concordia University, Montreal, QC H3G 1M8, Canada; alannah.gowling@mail.concordia.ca

**Keywords:** anion exchange ionomer, AEMFC, binder, alkaline fuel cell, electrode stability, electrochemical energy, functional polymers

## Abstract

Anion exchange ionomer (AEI) binders are critical to the performance of alkaline electrochemical devices (i.e., fuel cells, electrolyzers, and batteries), as they facilitate ion transport, provide structural integrity, and improve the overall performance and lifespan of these devices. These binders not only ensure ion transport but also provide mechanical stability to the electrode materials. Recently, there has been significant progress in designing AEIs that are more compatible with existing electrode materials and electrolytes. This review summarizes the different types of AEI binders, focusing on their chemical structure, functionalization, conductivity, and how they affect the performance of alkaline fuel cells, specifically, anion exchange membrane fuel cells (AEMFCs). It also discusses how factors like functional groups, polymer backbone and side-chain flexibility, and ion exchange capacity balance conductivity, mechanical strength, and water uptake (WU). Recent advances in material design, such as polymer blends, composites, and crosslinked ionomers, as well as electrode setup, such as asymmetric ionomer electrodes, are explored as methods for improving stability and ion transport. The main challenges facing AEIs, including water management, alkaline degradation, phase separation, mechanical robustness, and long-term durability, are discussed along with strategies for overcoming them. Finally, we outline future research directions for developing scalable, economical solutions and integrating these binders with new electrode materials to help improve the performance and stability of next-generation AEMFCs.

## 1. Introduction

Increased energy demands and population growth have led to heavy reliance on fossil fuels [1]. In 2016, the threat of climate change led to the sustainability goals of the Paris Agreement [2], which require extensive action towards carbon emissions reduction [3]. To achieve the reduced carbon emissions targets, governments have been taking part in initiatives such as the passing of laws and promoting the development of renewable energy devices [2,4,5,6]. Supporting the growing electrical grid demands requires a diverse and flexible network of reliable clean energy options [7,8]. Integrating clean energy sources, such as hydrogen, can overcome barriers caused by the intermittence of energy sources that rely on natural phenomena (e.g., solar, wind etc.) [9]. This renewable hydrogen is known as green hydrogen, which is generated from water electrolysis [10,11,12]. Renewable energy powers the electrolyzer to split water into hydrogen, an energy-dense fuel for chemical energy storage [11,12,13]. Green hydrogen has the potential to play a vital role in grid balancing, wherein excess renewable energy produced during peak periods can be stored long-term as hydrogen and later converted back to electricity when needed [13]. The transition of the global energy supply towards renewable energy sources has challenged energy technologies to further improve performance and bring devices to consumer markets [8,14]. Hydrogen-based electrochemical energy conversion devices (i.e., fuel cells) have gained interest for their potential to be a source of chemical energy [14,15,16], which can aid in the replacement of fossil fuels [1,8,14]. Recently, the anion exchange membrane fuel cell (AEMFC) is beginning to gain more traction relative to the proton exchange membrane fuel cell (PEMFC) due to the benefits of operating in alkaline conditions, particularly the ability to use non-platinum-group metal (non-PGM) as catalysts [14,15,16,17,18].

**Figure 1:**
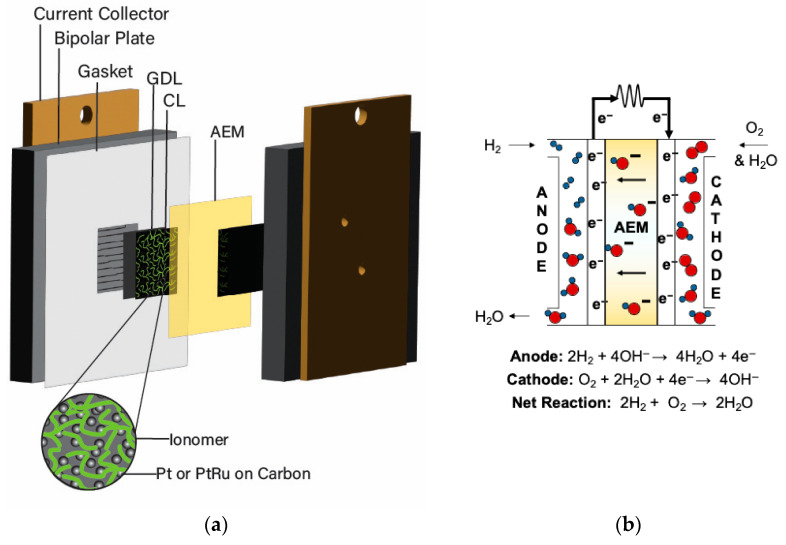
Schematics of the anion exchange membrane fuel cell (AEMFC) showing (**a**) major components and ionomer distribution within the catalyst layer (CL) and (**b**) an overview of the working principles. Hydrogen molecules are drawn in blue, while oxygen molecules are drawn in red.

AEMFCs comprise a membrane electrode assembly (MEA) compressed between bipolar plates, conducting plates, and end plates on either side (See Figure 1a). The main component of the AEMFC for electrochemical reactions and ion transport is the MEA, which contains an anode, anion exchange membrane (AEM), and cathode. The role of the AEM is to act as a solid electrolyte to facilitate the transport of hydroxide (^−^OH) between the electrodes [14,15,18,19]. Each electrode contains a gas diffusion layer (GDL) and a catalyst layer (CL) for facilitating the governing reactions. Within the catalyst layer there is a metal catalyst (e.g., Pt, PtRu) on carbon support material and an anion exchange ionomer (AEI) acting as a catalyst binder. A general overview of the AEMFC working principle can be seen in Figure 1b.

**Figure 2:**
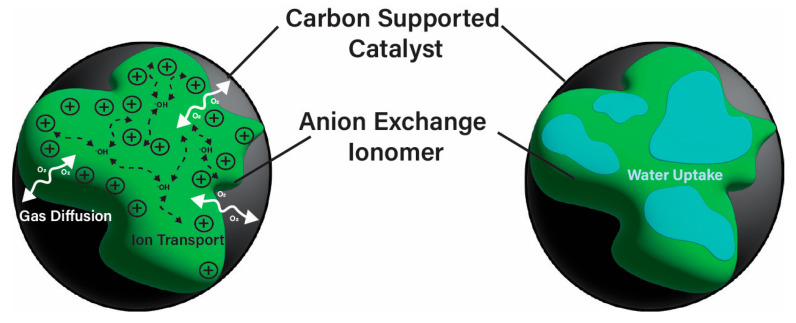
Schematic of an anion exchange ionomer (AEI) (green) bound to carbon-supported catalyst (black) with depiction of main functions within the catalyst layer (CL). Black arrows represent ion transport pathways (OH^−^ conduction) and gas diffusion (O_2_) to the catalyst surface. Blue domains indicate water uptake regions within the AEI.

Carbon support materials have a large surface area used for dispersion of the metal catalyst and are able to form pores for material movement that can affect the electrode structure along with the AEI [20]. AEIs act as a binder to the catalyst (and support material) that functions as a pathway for transport of materials (e.g., ions, reactant gas, water) within the CL, which can influence the overall AEMFC performance [21,22,23,24]. Similar to the AEM, the AEI is an ionic polymer that contains cationic components for interacting with the hydroxide anion (^−^OH) [25]. The cation components of the AEI form ionic channels that act as pathways (see Figure 2) to facilitate the movement of ^−^OH, often using a Grotthuss mechanism [26,27], between the catalyst active sites and AEM. The AEI is important for facilitating ion transport between the electrodes and AEM, as well as for formation of the triple-phase boundary layer [20] (i.e., the interface of catalyst, AEI, and gases [28]). The reactant gases utilize the AEI within the CL to reach the catalyst surface to react with the transported ions or other reactants [29]. As the AEI binds to the catalyst, it creates a network within the CL, which heavily influences performance parameters [25,30], wherein the morphology can be affected by the methods of fabrication [30]. Studies have been frequently focused on improving AEMs for AEMFC, while the specific influence of the AEI, as well as the fabricated electrodes and operating conditions, on performance-dictating parameters has historically received less attention and remains a significant knowledge gap [31].

Limited durability remains a major hurdle to the implementation of AEMFC technology, with water management being a critical factor. The operating conditions required to achieve desirable high current density result in system flooding that impacts the ability to operate for long durations [32]. In AEMFCs, water is produced at the anode during the hydrogen oxidation reaction (HOR), which can lead to flooding and mass transport limitations, especially when combined with humidified inlet gases [32,33]. Conversely, water is consumed at the cathode during the oxygen reduction reaction (ORR), often resulting in drying and degradation of the AEM and CL [32,33,34,35,36]. The contrasting water management challenges necessitates the need to assess each electrode to meet individual needs [33]. Aspects of AEMFC operation such as current density, temperature, relative humidity (RH), and CL porosity can have an influence on device water management [33]. The understanding of AEI properties is essential due to the important role it plays in AEMFC performance [37]. Properties of the AEI and its integration within the CL can be tailored to optimize the water balance across the AEMFC and improve overall performance. The cathode has a significant impact on AEMFC performance, and improvements within this electrode alone can allow for further technology advancement [29,37]. Under drier operating conditions, the degradation of the cathode AEI has been seen to show a more significant impact of voltage decays than AEM degradation [38]. IEC is often modified for increased water uptake (to reduce drying effects); however, this impacts mass transport in the CL, and therefore, its impact on the performance and gas permeability must be paid attention to [39]. Current reports of methods to improve AEI properties and electrode fabrication will be presented in this review. These methods include advancement of AEI polymer design, performance improvements through focused efforts on fabrication of the MEA, and water management to prevent anodic flood/cathodic drying that occurs due to AEMFC operation.

## 2. Design of Ionomers for Stability and Performance

The design of ionic polymers (i.e., AEMs and AEIs) with the properties necessary for high performance has been ongoing throughout the development of AEMFC technology. The property requirements of the AEI tend to differ from the AEM due to their distinct roles in the AEMFC [15,40]. While both the AEM and AEI require high ionic conductivity and excellent chemical stability in alkaline media, AEIs also prioritize strong binding to the catalyst and AEM, formation of ionic pathways via phase separation, permeability to reactant gases, and IEC and water uptake (WU), which are tailorable for the individual needs of each electrode [15,37,40,41,42,43,44]. Commonly used AEI polymers are listed in Table 1 and include the following classes: poly(phenylene oxide), poly(ethylene-c-tetrafluoroethylene), poly (aryl ether sulfone), poly(aryl piperidinium), poly(phenylene), poly(fluorene), poly(styrene), polynorbornene, and poly(benzimidazoliums) [15,43]. The most common cationic functional groups employed are trimethylammonium, n-methyl pyrrolidinium, n-methylpiperidinium, and imidazolium [15,41,43]. The benefits, challenges, and typical polymerization methods of these polymers have been summarized well in previous articles [15,40,45,46,47]. At this time, there are a select few of commercially available ionomers for use in alkaline electrochemical devices: Fumion^®^ (polyaromatic with a QA functional group), Aemion^+®^ (polyimidazolium), Sustanion^®^ (imidazolium-functionalized polystyrene), and PiperION^®^ (poly aryl piperidinium) [15].

In particular, there has been notable success in AEMFC performance with the use of polyfluorenes [24,35,48,49,50,51,52,53,54], poly (aryl piperidinium) [24,51,52,53,54,55,56], and polynorbornenes as AEIs and AEMs [24,36,57,58]. The 2000 h stable AEMFC of Ul Hussan and coworkers was achieved when using polynorbornene-based polymers [36], and the success of Bae and Kim and coworkers was found when utilizing FLNs for long-term stability [35,49]. Poly(ionic) liquids (PILs) are an emerging polymer class that have also been of increased interest due to their ideal stability and conductivity properties [59,60,61,62]. The optimization of AEIs has shown success in performance improvements and even more so when used in tandem with optimized operating parameters.

**Table 1:** Summary of benefits and challenges when utilizing common polymer classes in AEI design.

Polymer Backbone	Benefits	Challenges
poly(phenylene oxide)	Alkaline stability when quaternized [63] Versatile backbone for functionalization [64] Rigid polyaromatic backbone [41]	Prone to degradation via cleavage at the aryl ether [41,63,64,65] Degradation in AEMFC conditions when below critical hydration [66]
poly(ethylene-co-tetrafluoroethylene)	Chemical, thermal, mechanical stability [67] Produces free radicals for grafting [67]	Mechanically weakens and material degrades in long-term aqueous alkaline media [68,69] Concerns of fluorine safety [44] High water uptake leads to excessive swelling in CL [36,70]
poly(aryl ether sulfone)	Thermal and chemical stability [43,71,72] Phase separation improves with quaternization on pendant groups [71]	Prone to degradation via cleavage at the aryl ether under AEMFC operating conditions [43,44,63,71,72,73,74]
poly(aryl piperidinium)	Functional group embedded in backbone [75] Chemically stable [53]	Vulnerable to ring nucleophilic attack and Hofmann elimination in highly alkaline environments [75] Dimensional stability decreases at high IEC [53]
PiperION^®^	Cationic piperidinium groups can inhibit phenyl adsorption [24] Suitable as an AEI for high current density [76]	Hydrophilicity impairs gas diffusion and performance, as swelling can block pathways [24]
poly(phenylene)	Alkaline-stable [17,63] Aromatic backbones have good mechanical properties, conductivity, low water uptake [44]	Prone to phenyl adsorption [77] Hydroxide-conducting polymers often require complex synthesis (Diels–Adler) [17]
Fumion^®^	Low-cost, easily accessible, and used widely in studies [78] Suitable as an AEI for low-current-density operation [76]	Poor physicochemical stability [79] and electrochemical performance (at high current density) [76,79] Low room-temperature ionic conductivity [76]
poly(fluorene)	Non-rotating phenyl groups prevent adsorption [24,49,77] Tunable hydrophobicity [49] Long-term alkaline stability [35,48,49]	Concerns related to fluorine safety have led to preference for fluorine-free ionomers [44]
poly(styrene)	Aryl-ether free backbone, durable in alkaline environment [80] In a copolymer, provides mechanical and chemical stability to hydrophilic monomers [81]	Brittle and requires copolymerization or functionalization for flexibility [41]
polynorbornene	Minimal-to-no phenyl adsorption [24] Durable and alkaline-stable [36] High performance as an AEI [24,36]	Traditionally synthesized via Diels–Adler, which requires harsh reaction conditions [82]
poly(benzimidazolium)	Chemical resistance, mechanical and thermal stability [17,83] Durability via substitution of C2 aromatic ring [84]	Imidazolium is susceptible to ring degradation in alkaline devices [17,84,85] Synthesis is challenging due to rigid structure [83]
Aemion^®^, Aemion+^®^	High chemical stability in alkaline environment [38,86] Good conductivity and mechanical properties [86]	Iodide counter ion difficult to exchange [87] and challenging to make ink solution [88] Swelling in OH^−^ form can block gas (i.e., H_2_, O_2_) transport [87]
Sustainion^®^	High conductivity (dependent on hydration, temperature, and counter-ion) [89] Alkaline-stable [90]	Material more optimized for CO_2_ electrolysis [90,91] Polymer is less studied or understood compared to other commercial materials [89,92] Low water adsorption compared to Fumion^®^ and PiperION^®^ (as an AEI) [76]

### 2.1. Ionomer Durability Challenges

#### 2.1.1. Phenyl Adsorption

**Figure 3:**
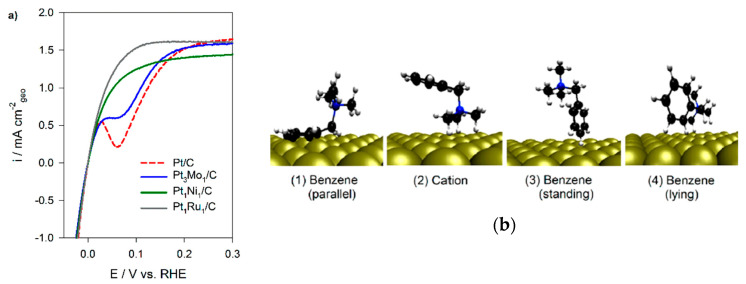
Effects of adapting for phenyl adsorption on performance. (**a**) HOR voltammograms of Pt alloy/C catalysts in 0.1 M BTMAOH at 25 °C, 900 rpm, and 5 mV s^−1^. Matanovic et al. [96], reprinted (adapted) with permission [96]. Copyright © 2017, American Chemical Society. (**b**) Phenyl interactions with the Pt(111) surface from DFT-optimized structures of BTMA. Matanovic et al. [96], reprinted (adapted) with permission [96]. Copyright © 2017, American Chemical Society.

Eliminating sources of instability is vital to achieving high performance and durability in alkaline electrochemical devices [24]. AEIs can play an important role in determining device stability. AEIs require low catalyst adsorption and high oxidative stability [55]. Incorporation of phenyl groups in the ionomer backbone has allowed for increased solubility in organic solvents and higher hydroxide conductivity [24,41]. However, adsorption of phenyl groups onto the Pt catalyst surface impedes active sites, leading to a significant decline in activity [24,84,93,94,95,96]. Kim and coworkers found that ionomers with a phenyl group positioned parallel to the Pt catalyst led to this surface adsorption and weakened HOR catalyst activity (Figure 3a,b) [96]. Density functional theory (DFT) calculations were used to explain the difference in the HOR activity between Pt and Pt-based bimetallic catalysts. In particular, they report quantitative adsorption energies for phenyl (benzene) fragments on Pt(111), identifying a flat-lying π-bonded configuration that competes with H adsorption and suppresses HOR activity; alloying with Ru weakens this interaction and mitigates inhibition. Although adsorption barrier calculations for benzene/Pt exist in the broader surface science literature, such analyses have not yet been widely applied to AEI–catalyst systems, where adsorption energies currently provide the most relevant mechanistic evidence to complement experimental findings. Further studies from Kim et al. found that phenyl adsorption effects were reduced or prevented by utilizing materials such as a metal alloy catalyst (i.e., PtRu) in the anode to weaken adsorption, as well as utilizing AEIs with aryl-ether-free backbones, low phenyl content, and/or cationic functional groups that competitively adsorb onto the catalyst surface [63,95,96]. Incorporating non-rotating phenyl groups in the backbone of polyfluorene-based polymers (FLNs) in the works of Bae et al. showed significantly improved AEMFC performance (>1 W cm^−2^), as the phenyl group was unable to rotate into the parallel position [63,77,94]. Challenges with phenyl adsorption in the HOR electrode have indicated the need for non-PGM catalysts as they can remove the Pt adsorption concerns entirely while decreasing catalyst expenses [63,94].

#### 2.1.2. Degradation Reactions

In addition to phenyl adsorption, other sources of degradation and instability occur in AEMFC polymers. Alkaline degradation impairs the ability to achieve long-term stability via unrecoverable performance losses [22,75,97]; therefore, it is an important consideration in AEI design. Aryl ethers are prone to degradation and cleavage mechanisms in alkaline environments, which has led to the development of aryl-ether-free ionomers [42,47,98]. Common QA polymer degradation mechanisms include SN2 nucleophilic substitution, elimination degradations (Hofmann, E2), ring-opening nucleophilic attacks, and oxidative radicals in strong alkaline environments inducing degradation [42,75,97,99]. QA-type ionomers have been found to be more alkaline-stable when tethered to an alkyl than when situated near oxygen atoms or when benzyl-substituted [99]. Li and coworkers reported that performance losses during a stability test (60 h, 0.6 A cm^−2^) were associated with the significant QA degradation of the AEI (QA poly(N-methyl-piperidine-co-p-terphenyl) (QAPPT)) and water management challenges [97]. The radicals of the ORR and applied cell potentials increased cathode degradation to be higher than the anode [97]. Ul Hassan and coworkers assessed the voltage losses of long-term AEMFC operation (3600 h, 0.6 A cm^−2^), which occurred at decay rates of 15 uV/h (0–2000 h), 118 uV/h (2000–2600 h), and 26 uV/h (2600–3600 h) [100]. While water rebalances throughout operation recovered some losses, most were unrecoverable due to degradation, primarily in the cathode [100].

Alkaline stability analysis of polyphenylene oxide (PPO) backbone with pyrrolidine cations and alkyl spacers was performed by Cao et al. using ^1^H NMR and FTIR spectroscopy of samples [25]. The cation salts (benzyl-pyrrolidine) exposed to 80 °C and 2 M KOH under constant voltage (1 V) showed complete degradation (i.e., no visible peaks); however, it was stable with an alkyl chain (N-methyl-1-heptylpyrrolidine salt) [25]. The pyrrolidine cation peaks of the polymer in 2 M NaOH did not show signs of significant Hofmann β elimination degradation after both 192 h and under constant 1 V at 80 °C for 96 h [25]. Thermal degradation indicated decomposition of PPO 441 °C due to backbone degradation at the C-O bonds, which decreased to 231 °C with the addition of pyrrolidine [25]. While the temperatures were above typical AEMFC operation [25], this indicates the impact of degradation.

Sen et al. studied AEI degradation using styrene-based PILs with N-heterocyclic cations of varied size in 1 M KOH/D_2_O for 4 weeks and analyzed them via ^1^H NMR [101]. The largest ring size (methylazonanium, nine-membered ring) displayed a 22.2% degree of degradation, which was predicted to be caused by the increased ring strain favoring elimination or substitution reactions [101]. The eight-membered ring (methylazocanium) showed some degree of degradation (2.2%), while five-, six-, and seven-membered rings (methylpyrrolidinium, methylpiperidinium, and methylazepanium, respectively) did not show signs of degradation, likely due to their lower ring strain [101]. Tipp and coworkers reported the use of correlation analysis and computational methods to determine the likelihood of (benz)imidazolium to experience ring-opening nucleophilic addition–elimination degradation reaction at the C-2 [85]. Through this study, it was found that the Gibbs free energy has a strong correlation to the hydroxide attack at C-2 and correlates with the Gibbs free energy of the ring-opening reactions [85]. This study highlighted the benefits of utilizing computational methods to assess the potential stability of ionomer materials [85].

### 2.2. Modifications to Ionomer Structure and Copolymerization for Improved Performance

#### 2.2.1. Conformational Changes to Polymer Backbone

**Figure 4:**
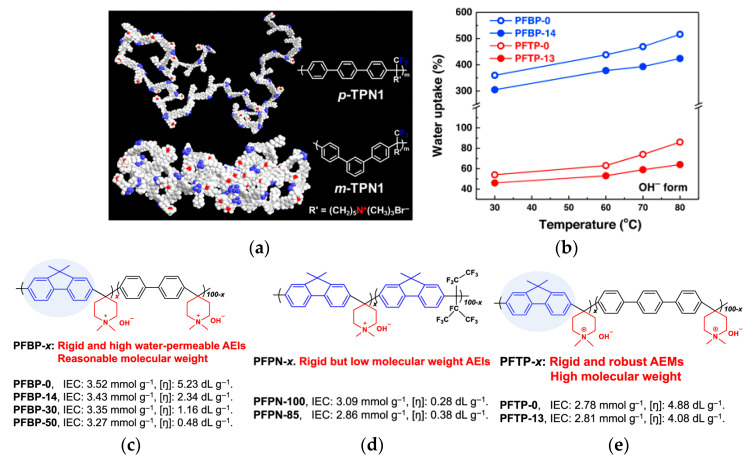
(**a**) Three-dimensional models showing backbone comparison of para-TPN1 and meta-TPN1 presented by Lee et al. [102]. Adapted with permission [102]. Copyright © 2017, American Chemical Society. (**b**) PFBP, PFPN, and PFTP molecular structure with their respective IEC and intrinsic viscosity [54]. Adapted with permission [54]. Copyright © 2021, *Nature Communications* (licensed under CC BY 4.0), Creative Commons. Resulting WU properties due to fluorene content and phenyl backbone changes in copolymers (**c**) PFBP, (**d**) PFPN, and (**e**) PFTP [54]. Adapted with permission [54]. Copyright © 2021, *Nature Communications* (licensed under CC BY 4.0), Creative Commons.

Analysis of ionomer performance has led to the use of further modifications in efforts to improve properties and AEMFC performance. The polymer backbone geometry has the ability to impact AEMFC performance [16]. Lee and coworkers reported that changing conformations from a para-TPN1 to meta-TPN1 (Figure 4a) increased flexibility, which improved backbone folding for phase separation [102]. The aryl-ether-free backbone and alkyl chain on the functional group allowed for alkaline stability (30 days, 1 M NaOH) in both conformations [102]. Though the meta-TPN1 and para-TPN1 were used as an AEM in applications for the study [102], the benefit of an increase in flexibility is applicable to AEI development. Hyun and coworkers utilized the backbone flexibility of meta-TPN1 and a trimethylammonium (TMA) cation to improve AEI–catalyst interactions [103]. Similar findings were reported by Zheng and coworkers, wherein a meta-QAPPT improved the AEI–catalyst interactions compared to a para-QAPPT due to the increased flexibility [23].

#### 2.2.2. High-Performing Poly(fluorenes) and Piperidinium Copolymer

Poly(fluorenes) (FLNs), as developed by Bae and coworkers (Table 1) [48], have been used as an AEI in AEMFC studies [63]. Maurya and coworkers found that the low phenyl adsorption and improved IEC of FLNs allowed for higher AEMFC performance (1.46 W cm^−2^) in comparison to ionomers with rotating biphenyls [63]. These FLNS have been incorporated as AEIs for further studies into AEMFC performance optimization [49,50,54,63,94,104]. For example, Chen and coworkers synthesized copolymers of FLNs with poly aryl piperidinium and varied the fluorene content to modify water permeability properties for AEIs and AEMs (Figure 4b–e) [54]. Their poly(fluorene-co-biphenyl N,N′-dimethyl piperidinium) with 14% fluorene (PFBP-14) had high AEMFC performance (2.34 W cm^−2^) and increased water permeability (Figure 4c) that allowed for operating at moderate–low RH for water management [54,104].

Building on this success, the same group explored alternative copolymer backbones to further optimize phase separation and hydration behavior while retaining the piperidinium cation. They developed poly(diphenyl-terphenyl N,N-dimethyl piperidinium) (PDTP-x) copolymers with varying ratios of diphenyl to terphenyl [55]. The diphenyl and alkyl spacers increased the size of hydrophilic channels, improving phase separation and WU [55,104]. The highest-performing diphenyl-to-terphenyl ratio of 75 (PDTP-75) performed better in an asymmetric PFBP-14/PDTP-75 (anode/cathode) configuration than a symmetric one, as the increased WU of PDTP-75 prevented drying in the cathode but was excessive for the anode [55]. Optimized AEMFC operating conditions allowed for a peak power density (PPD) increase to 2.08 W cm^−2^ with the PFBP-14/PDTP-75 configuration and to 2.58 W cm^−2^ with symmetric PFBP-14 [55]. Chen and coworkers subsequently utilized the increased water permeability of PFBP-14 as an AEI to retain 70% of the peak power density achieved at 1000 mL min^−1^ H_2_ flow (2.42 W cm^−2^) when operated at a more cost-effective 75 mL min^−1^ flowrate (1.77 W cm^−2^) [104]. These results demonstrate that targeted structural modifications to AEIs, such as fluorene content and asymmetric pairing, enable effective water management and sustained performance even under reduced hydrogen flow, supporting the development of more efficient and cost-effective AEMFC systems.

In the further interest of utilizing fluorenyl groups for alkaline device applications, Ono and coworkers synthesized and studied a material using partially fluorinated alkylene main chains that contained fluorenyl groups with pendant trimethyl ammoniums (QPAF-4) for anion conduction [105]. The copolymer design resulted in a phase separation dependent on the hydrophobic perfluoroalkylenes rather than the hydrophilic ammonium groups [105]. Small-angle X-ray scattering (SAXS) showed a prominent peak associated with the hydrophilic domain spacing, with d ≈ 8 nm at 30% RH that shifted slightly to ca. 9 nm at 90% RH [105]. While the study focused on material development for AEMs primarily, it was utilized as well as an AEI in this study for an alternative alkaline device (hydrazine alkaline FC) [105] and in AEMFC applications in a subsequent study by Koronka et al. (performance summarized in Table 2) that modified the AEM to have a piperidinium pendant [51]. Wang et al. reported a poly(aryl piperidinium)-based ionomer for AEMFC applications that incorporated 2,2,2-trifluoroacetophenone for tuning the IEC and maintaining alkaline stability of piperidinium cations and biphenyl groups (PAP-BP-x) [106]. The use of biphenyls allowed for the overall polymer to be soluble in the IPA/H_2_O solvent used for catalyst ink dispersions, which is ideal for CL fabrication [106]. The use of the aryl-ether-free backbone was beneficial for preventing catalyst adsorption and common degradation mechanisms in alkaline environments [106]. In addition to AEMFC performance tests (highest performance summarized in Table 2), a long-term stability test showed it was stable for 300 h at 0.5 A cm^−2^ and with H_2_/air (CO_2_-free) [106]. The use of air is beneficial for advancement at the laboratory scale, as using air in place of pure O_2_ can be more ideal for scale-up in terms of eventually reaching the ultimate goal to use ambient air for operation, which is needed for practical application of AEMFCs [107].

Despite the inconsistent standard testing across the samples in the above table, the performance can be used to highlight the benefits of FLN and PAP copolymerization. Considering the PAP-BP and FLN samples as the co-polymer components on their own, unlike the PFBP copolymer, neither exceeded 1 W cm^−2^ PPD. Though the QPAF-4 materials were found to be durable [51,105], the performance of the material as an AEI was not as high as the PFBP copolymer. The incorporation piperidinium groups on QPAF in place of TMA showed success as an AEM [51] and could be considered a beneficial modification to the AEI.

#### 2.2.3. Alkyl Spacers and Sidechains

In addition to backbone modifications, tuning the sidechain architecture—such as the use of alkyl spacers—offers another strategy to balance ion conductivity, water uptake, and dimensional stability in AEIs. The increased length of an HTMA sidechain incorporated onto poly(dibenzyl-co-terphenyl piperidinium) (s-PDTP) AEI (Table 3) was reported by Kim and coworkers to increase the IEC and WU, which similarly improved performance and phase separation [108] relative to a similar structure without the alkyl spacer (PDTP-50) [55,108]. The moderate IEC (3 mmol g^−1^) had the highest PPD (s-PDTP-65, 0.95 W cm^−2^) compared to the low IEC (2.5 mmol g^−1^) and high IEC (>3.4 mmol g^−1^) (0.75 W cm^−2^ and 0.8 W cm^−2^, respectively) [108]. This was attributed to low ion conductivity in lower IEC AEIs and lower dimensional stability with higher IEC AEIs [108]. Optimizing the operating conditions through backpressure further increased PPD to 1.47 W cm^−1^ [108]. A comparison of PDTP with and without alkyl spacers can be seen below in Table 3.

The use of alkyl spacers to improve performance and mitigate catalyst adsorption for a PPO-based AEI with a pyrrolidinium (Py) cation and varied terminal alkyl groups (4, 7, and 10 carbons) was reported by Cao et al. [25]. Analysis of the ORR indicated that the alkyl spacer improved electrochemical activity through positive shifts in linear sweep voltammetry (LSV) curves, with the highest CL ECSA (62.86 m^2^ g^−1^) being that of the 7 carbon terminal alkyl (PPO-7-Py7) [25]. AEMFC test comparisons utilizing AEI PPO-7Pyn with *n* = 4, 7, and 10 terminal alkyls and a QPPO AEM were conducted [25]. The PPO-7Py7 ionomer had the best PPD (0.261 W cm^−2^) compared to the PPO-7Py4 (0.243 W cm^−2^) and PPO-7Py10 (0.217 W cm^−2^) [25]. EIS analysis under OCV indicated that PPO-7Py7 had the lowest ohmic resistance (0.028 Ω cm^2^); however, it was comparable to PPO-7Py4 and PPO-7Py10 (0.032 and 0.062 Ω cm^2^, respectively) [25]. The low non-ohmic resistance (CL-AEM contact resistance) and charge transfer resistance contributed to the improved PPO-7Py7 performance [25]. The MEA was not stable for long-term operation despite the AEIs maintaining electrochemical activity. Voltage drops indicated AEM swelling and flooding effects under full RH, especially after 12 h [25]. These results further highlight the link between AEI chemical structure and water management.

The use of alkyl spacers in AEI development has allowed for improvements in material properties, as seen in the presented samples. The addition of alkyl spacers is not limited to the ionomers presented in this section. The structure of previously presented samples such as FLNs contain alkyl spacer chains, and it can be noticed in samples of subsequent sections that such chains have been incorporated into their structure. The benefits of this AEI design modification can be used in addition to other methods to further optimize the performance of ionomer material.

#### 2.2.4. Ionomer Microphase Separation

Alterations to the chemical structure of ionomers to improve microphase separation and phenyl adsorption reductions have been explored (Table 3). Sun and coworkers used the addition of ortho-TPN in poly(norbornane-co-aryl piperidinium) (PDPN) to form more compact folding, while the norbornane offered low phenyl adsorption [16]. SAXS analysis was used to identify the phase separation of the hydrophobic and hydrophilic regions [16]. The hydrophilic phase width increased with the DPN incorporation [16]. PDPN-26.0 exhibited a hydrophilic phase width of 10.2 nm and a hydrophilic area ratio of 44%, whereas the poly(terphenyl) control (PTP) showed 7.3 nm and 52%, respectively [16]. Consistent with this morphology trend, OH^−^ conductivity at 80 °C increased from ~134 mS cm^−1^ (PTP) to ~144 mS cm^−1^ (PDPN-10.7) and ~157 mS cm^−1^ (PDPN-19.3) before dropping to ~86.5 mS cm^−1^ for PDPN-26.0 due to excessive swelling. The distinct phase separation was further verified when visualized using transmission electron microscopy (TEM) [16]. The 2,3-diphenylbicyclo(2.2.1)heptane (DPN) of this copolymer improved microphase separation, while the WU and swelling ratio (SR) increased in accordance with the DPN quantity due to its hydrophobicity and twisted structure favoring microphase separation [16]. Steric hinderance limited molecular weight with increasing DPN content, such that the highest DPN content (26%, PDPN-26) was unsuccessful as an AEM [16]. Despite the limited molecular weight, the high DPN content of PDPN-26 was the best-performing of the AEIs paired with a PDPN-19.3 (19.3% DPN) AEM [16]. Gokulapriyan et al. utilized poly N-aryl piperidinium (PNAP) with slight variation in added *para*-TPN content (0.5 eq or 1 eq) to create PNAP-1 (0.5 eq) and PNAP-2 (1 eq) for improved alkaline stability [75]. PNAP-2 had ideal WU, SR, and ORR activity (e.g., low Tafel) for use as an AEI compared to PNAP-1 [75]. The structure of the para-terphenyls and 9,10-diphenylanthracene groups allowed for the free volume needed for good oxygen transport within the CL [75]. Atomic force microscopy (AFM) was used to analyze the microphase separation [75]. The hydrophilic phase width was found to be 107 nm for PNAP-1 and 122 nm for PNAP-2 [75]. The increase in para-TPN resulted in developed hydrophilic channels for improved conductivity [75]. The anthracene strengthens rigidity in the PNAP backbone to develop microphase separation for improved conductivity and stability and, subsequently, the performance (2.07 W cm^−2^) when utilized in the MEA during fuel cell tests [75]. The conformational change in switching from a para-TPN1 to a meta-TPN1 displayed improved phased separations and morphologies [102]. SAXS analysis indicated that while the para-TPN1 showed low peak intensity (i.e., insignificant phase separation), the meta-TPN1 showed two distinct peaks with a *d* spacing of 6.5 and 5 nm [102]. This indicated the folding due to the backbone conformational change allowed for phase separation to occur [102]. These studies demonstrate that precise tuning of ionomer structure, including backbone rigidity, steric bulk, and substituent positioning, can enhance AEI performance by promoting microphase separation.

The structure modifications in the backbone allowed for building upon the beneficial characteristics of these ionomer materials. The case of PDPN indicates how ionomer materials that are unable to form a film may still have great potential within the CL. Previous examples, such as those outlined in Section 2.2.3, have also looked to improve phase separation as part of the material development. Phase separation can aid in decreasing the mass transport resistance and impeded gas diffusion to the catalyst caused by flooding effects of highly hydrophilic (high IEC) AEIs [24]. Within Table 4, QP-NB and *meta*-TPN1 are included as additional modifications that were utilized to reduce phenyl adsorption, which will be discussed further in the following section.

#### 2.2.5. Further Modifications for Phenyl Adsorption Decreases

**Figure 5:**
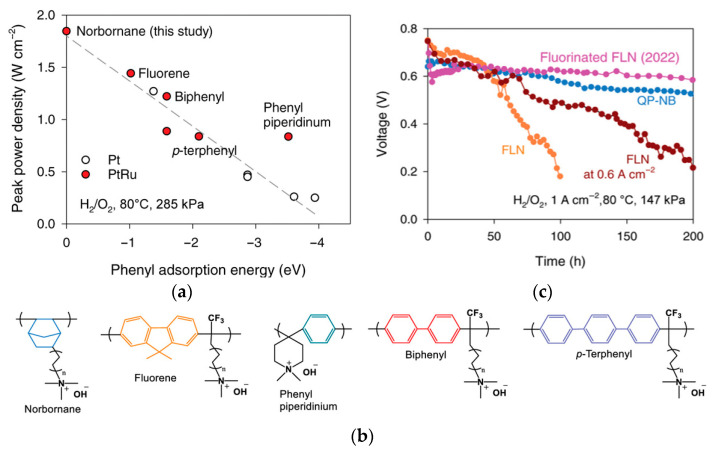
(**a**) Phenyl adsorption and PPD correlations between AEI changes under identical AEM and operating conditions. Adapted from Leonard et al. [24]. Copyright © 2022, the authors [24]. *Advanced Energy Materials*, published by Wiley-VCH Gmb (licensed by CC BY-NC 4.0). (**b**) Structures of backbone types, adapted from Leonard et al. [24]. Copyright © 2022, the authors [24]. *Advanced Energy Materials*, published by Wiley-VCH Gmb (licensed by CC BY-NC 4.0). (**c**) Comparison of voltage change over time at a constant density with FLN AEIs and QB-NB. Adapted from Leonard et al. [24]. Copyright © 2022, the authors [24]. *Advanced Energy Materials*, published by Wiley-VCH Gmb (licensed by CC BY-NC 4.0).

Building on prior efforts to reduce phenyl adsorption through sidechain design, additional strategies have focused on backbone and functional group selection to further enhance AEI performance. Leonard et al. explored the effects of phenyl adsorption energy with commonly used ionomers (*meta*-TPN, PiperIon^®^, FLN, polynorbornene) and the potential of phenyl-free polynorbornenes (QP-NB) to improve performance [24]. AEMFC performance was compared with varying AEIs (*para*-TPN, biphenyl, and FLN [77], QP-NB, phenyl piperidinium while employing the same AEM (HTMA-DAPP) and identical operating conditions; as shown in Figure 5a, performance clearly improved with decreasing phenyl adsorption [24]. Long-term stability tests (Figure 5c) showed the importance of optimizing the operating conditions for the AEI and water management [24]. QP-NB had high alkaline stability for 1000 h in 1 M NaOH at 80 °C, and at its optimal operating conditions, performance reached 1.41 W cm^−2^ [24]. These results reinforce the value of minimizing backbone phenyl content and highlight the importance of matching AEI chemistries with appropriate operating regimes to fully realize performance benefits.

#### 2.2.6. Functionalized Polystyrenes

Polystyrene (PS) has been incorporated to develop AEIs for AEMFC applications, as summarized in Table 4. Chen and coworkers reported the use of γ-amine-piperidinium-functionalized PS polymers (P-AP-Ca), which contained carbazole that was varied in content for IEC alterations and N-heterocyclic ammonium (NHA) for its high alkaline stability [110]. Without the carbazole group (P-AP), the IEC and subsequent WU and hydroxide conductivity were reported to be high, while addition of carbazole (P-AP-CA-10 and P-AP-Ca-22) resulted in decreases in these properties [110]. The alkaline stability of PS and NHA in P-AP allowed for a reported 1700 h stability in 80 °C NaOH without degradation [110]. P-AP was the highest-performing of the AEI samples, achieving a PPD of 1.35 W cm^−2^ with a Co-Mn cathode and 1.63 W cm^−2^ with a Pt cathode, exceeding the common commercially available polymers tested in similar operating conditions [110]. The P-AP was reported to withstand running at 0.3 A cm^−2^ 65 °C for 140 h at lower gas flowrates [110], indicating potential for durable and economic AEMFC use.

Chae and coworkers also reported on a PS-based AEI, varying the benzyl sulfonyl chloride content that was functionalized with trimethyl ammonium (SxxQA30-C6), highlighting the influence of molecular weight and alkyl chain length on AEMFC performance [80]. Increasing the styrene-to-benzyl sulfonyl ratio and the subsequent overall molar mass increase resulted in agglomeration within the catalyst ink that translated to the CL of MEAs [80]. The highest-molar-mass AEI (S63QA30-C6) (1000 styrene units–benzenesulfonyl chloride) thus had poor performance due to the non-uniform CL, while the lowest-molar-mass AEI (S10QA30-C6) (65 styrene units–benzenesulfonyl chloride) was not able to form adequate bonds with the catalyst [80]. The most ideal ratio of the samples was the medium-molar-mass AEI of 300 styrene units to −1 benzenesulfonylchloride (S29QA30-C6), which had the lowest ohmic resistance (0.0897 Ω cm^2^) and the ability to form required ionic channels for ideal AEMFC function [80].

Several groups [58,69,111,112,113] have reported high AEMFC performance while utilizing similar electrodes containing a vinyl benzyl chloride (VBC)-grafted ETFE AEI functionalized with trimethylamine, synthesized by the Varcoe group (Table 5) [112]. For example, the highest reported PPD for AEMFC tests to date was achieved in 2019 by Huang et al. [58] at 3.4 W cm^−2^ using ETFE-*graft*-PS-based ionomers. Yang-Neyerlin et al. later used the same ETFE-*graft*-vinylbenzyltrimethylammonium chloride (ETFE-*g*-VBTMA Cl^+^) as an AEI paired with polysulfone-poly(diallylpiperidinium hydroxide) (PSF) AEMs of varying IEC, as reported in [113]. AEMFC performance varied with the AEM IEC in terms of kinetic and mass transport losses, cathode structural changes, and ECSA (anode/cathode AEIs remained consistent) [113]. Investigation into the electrodes after AEMFC operation indicated that the cathode experienced carbon corrosion, agglomerates, and particle growth, which caused performance degradation, while the anode did not [113]. The extent of the CL structural changes were moderate in the cathodes of the more stable MEAs and more extreme in the low-performing MEAs [113]. The best-performing MEA (with AEM of IEC 1.3) experienced the lowest overpotentials (kinetic, ohmic, mass transport), CL structural changes, and performance degradation (5% after 540 h) [113]. The PPD results and operating conditions are summarized in Table 5 [113]. The difference in CL structural changes emphasized the need to further investigate the water management, catalyst choice (Pt vs. PtRu), and resistance for a better understanding of their impacts on electrode performance [113]. Furthermore, the impact of the AEM-CL interactions identified the need to consider this relationship in assessing AEI and optimizing its AEMFC performance. Overall, PS-based AEIs have shown strong durability and peak performance in AEMFCs, but their effectiveness depends on balancing functional group incorporation, optimizing molecular weight, and managing electrode interactions to avoid performance losses and cathode degradation.

#### 2.2.7. Poly(Ionic Liquids)

Poly(ionic liquids) (PILs) are polyelectrolytes containing an ionic liquid within the monomer repeat units. PILs have become an emerging class of polymers for use in fuel cells as they offer high chemical and thermal stability, freedom in structural modifications and polymerization methods, and low environmental impact [15,60,61,62,114,115]. The incorporation of covalently attached cationic functional groups (e.g., pyrrolidinium, imidazole, pyridinium, etc.) into PILs has allowed for their use as single ion conductors [61,115]. Copolymerization allows for the combination of mechanical properties of non-ILs with the conductivity of the PIL and overall polymer property tuning [61,115]. Multi-block copolymerization can be used to improve phase separation, where block copolymers (BCPs) with hydrophilic/hydrophobic regions can lead to self-assembled morphologies that vary depending on PIL content (e.g., bi-continuous gyroid, lamellae, and cylinders) and improved ionic conductivity over random copolymer (RCP) configurations [61,81,114,116,117,118,119]. A summary of PILs utilized as AEIs is given in Table 6.

**Figure 6:**
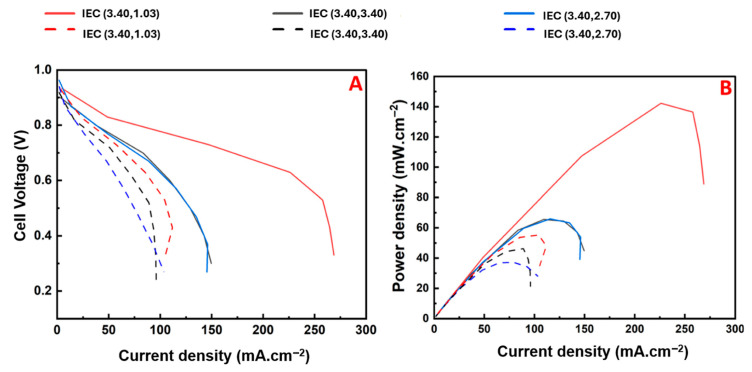
(**A**) Polarization curves and (**B**) performance curves initially (dashed lines) and after 18 h of operation (solid lines) with 100% RH anode/cathode. IEC (cathode, anode) utilizing PDADMAC-b-PS AEIs. Obtained and adapted from Shubair et al. [118] with permission. Copyright © 2025, the author(s). Published on behalf of The Electrochemical Society by IOP Publishing Limited. (Licensed under: CC BY 4.0).

Shubair et al. synthesized BCPs of polydiallyl dimethyl ammonium chloride (PDADMAC) and polystyrene (PS) (via a RAFT polymerization protocol developed by Cotanda and coworkers [81]) alongside RCP analogs of the same polymers [118]. These BCPs were utilized as AEIs because of their highly hygroscopic PIL unit as a means to modify WU/IEC to improve AEMFC water management (Figure 6) [118]. This study found that performance improvements could be achieved by altering AEI configurations alone, wherein a high-IEC (i.e., 3.40 mmol g^−1^) AEI was consistently utilized at the cathode to prevent dry-out while the AEI IEC was varied at the anode (i.e., 1.03, 2.70, 3.40 mmol g^−1^). Ultimately, as shown in Figure 5, the power density was more than doubled when switching from a typical symmetric AEI configuration (i.e., using an AEI of IEC 3.40 mmol g^−1^ at the cathode and anode) to an asymmetric AEI configuration, wherein the lowest-IEC AEI was employed at the anode to prevent flooding [118]. These findings highlight the versatility of PIL-based AEIs and that asymmetric configurations alone can significantly enhance AEMFC performance.

In PILs developed by Nykaza and coworkers, the alkyl spacer between the cation (butylimidazolium) and polymer backbone increased from two carbons in 1-[(2-methacyloyloxy)ethyl]-3-butylimidazolium bromide (MEBIm-Br) to eleven carbons in 1-[(2-methacyloyloxy)undecyl]-3-butylimidazolium bromide (MUBIm-Br) [114]. The PIL was utilized in a BCP with methyl methacrylate (MMA) to develop poly(MMA-b-MUBIm-HCO_3_). SAXS analysis revealed that one of the long-chain PIL BCPs (40.2 vol% PIL) formed lamellar domains, evidenced by reflections at q*, 2q*, and 4q*, with a Bragg spacing of 27.9 nm [114]. Similarly, the short-chain PIL BCP (39.1 vol% PIL) exhibited a lamellar morphology (q*, 2q*, 3q*, 4q*) with a slightly smaller spacing of 25.1 nm. In contrast, the long-chain PIL BCP with a higher ionic content (59 vol% PIL) produced cylindrical domains (q*, 2q*) due to the increased volume fraction of the PIL block. Importantly, these morphological differences were reflected in the ionic transport: the lamellar samples with well-defined phase separation exhibited hydroxide conductivities up to ~2 × 10^−2^ S cm^−1^ at 120 °C, whereas the cylindrical BCP showed lower conductivity despite its higher PIL fraction. This demonstrates that domain spacing and long-range-ordered morphology are as critical as the ionic content for achieving efficient ion transport in PIL-based BCPs [114]. The performance of the long-chain PIL BCP as an AEI and AEM was assessed with different fabrication techniques and backpressure [117]. AEMFC single-cell tests with poly(MMA-b-MUBIm-HCO_3_) (20 mol% PIL) showed a performance of 0.0293 W cm^−2^ when the backpressure was increased to 172 kPa, which was an improvement compared to the performance with no backpressure (0.0202 W cm^−2^) [117].

PILs have also been utilized in recent studies regarding AEI–catalyst interactions and the use of non-PGM catalysts. Gokulpriyan and coworkers incorporated an imidazolium IL into quaternized poly(2,6-dimethyl-1,4-phenylene oxide) (QPPO) to create composite QIL for improved conductivity and WU properties as an AEM and AEI [120]. Morphology analysis displayed distinct phase separation between the hydrophilic ILs, QA, and the hydrophobic backbone [120]. The hygroscopic properties of the IL increased water absorption (57–82%) as well as the conductivity at 80 °C [120]. QIL-8 (8% IL) had the highest IEC and hydroxide conductivity of the samples (3.3 mmol g^−1^, 135 mS cm^−1^, respectively), while surpassing 8% IL decreased these values due to excess WU [120]. The QIL was found to have improved alkaline stability in comparison to QPPO, likely due to the steric hinderance of the IL preventing nucleophilic attacks [120]. Analysis of the ORR showed that the QIL-8 had a lower Tafel (58 mW dec^−1^) compared to QPPO (71 mV dec^−1^) with an NIF cathode catalyst [120]. Favero and coworkers utilized PILs as a solution to aggregation in electrode fabrication [21]. The study compared Fumion^®^ to a synthesized copolymer of polystyrene and vinyl imidazole with anion poly(St-co-VImNTF2) and used a single-site iron catalyst iron phthalocyanine (Fe(II)PC) as the cathodic catalyst [21]. The PILs were an ideal candidate for an AEI, as the active sites of the catalyst were well dispersed within ink solutions and aggregates were prevented due to its presence [21]. The PIL improved oxygen transport in comparison to Fumion^®^ due to decreased transport resistance and higher ORR activity [21]. The hydrophobic styrene within the PIL was reported to have improved OH destabilization and thus increased the catalyst activity within the GDE [21]. The development of ionomers for AEMFCs has progressed beyond traditional property optimization, such as conductivity and stability, to include deeper analysis of the role of AEI in CL interactions, ink formulation, and water management. These aspects of AEI design, along with strategies for tuning fabrication methods and operating conditions, are explored in the following sections.

## 3. Fabrication of AEMFC Catalyst Layer, Ionomer Catalyst Binders

Ionomers utilized as catalyst binders (i.e., AEIs) have significant impact on the overall AEMFC performance [98]. The ionomer selected and its quantity are important considerations for the fabrication of electrodes [87,121,122]. Improving water management (e.g., preventing anodic flooding and cathode dry-out) has received increasing attention recently owing to its importance in long-term performance stability, while the contributions of an optimized CL morphology can aid in improving overall performance [24,28]. The CL structure is an important design parameter that can influence the water management challenges often found in AEMFCs [103]. The improved utilization of ionomers through optimizations in fabrication of the CL, ionomer–catalyst relationship, and influences on morphology has recently become an area of interest [103,123] owing to its ability to improve performance.

**Figure 7:**
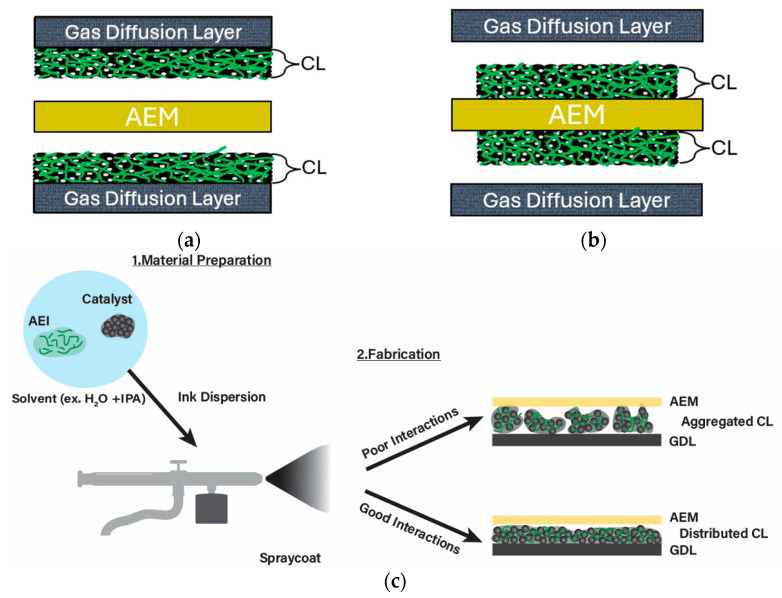
Depiction of (**a**) catalyst-coated membrane (CCM) and (**b**) catalyst-coated substrate (CCS). (**c**) Depiction of fabricated catalyst layer (CL) with good AEI–catalyst–solvent interactions (distributed CL) and poor AEI–catalyst–solvent interactions (aggregated CL).

Morphology can be influenced by the interactions between CL components, preparation methods, and quantity of AEI/catalysts [20]. The electrode and CL morphology is important in AEMFC function, as it can affect the ability to form transport channels and porosity structures for materials such as water, reactant gas, and ions [20,28,121]. A goal of anode/cathode fabrication is to optimize the electrochemically active site (electrochemical surface area, ECSA) in the CL to improve AEMFC performance [28]. The effects of solvent choice on the fabricated CL can differ with alkaline ionomers, since it is often required to use other (organic) solvents to aid in dispersing ionomers in IPA/water, unlike with common PEMFC ionomers (e.g., Nafion) [30]. The surface tensions resulting from ionomer–catalyst–solvent interactions can vary the morphology within the ink dispersions prior to CL fabrication [30] (Figure 7c). Unoptimized ink dispersions have been found to cause aggregation, which can negatively impact CL morphology, material transport, and overall performance [20,21,23,28,30,84,87,121,122,123,124,125,126]. The tendency to aggregate can depend on the solvents and their electrostatic repulsive forces against surface tensions of dispersion contents [30]. Additionally, CL preparation using spray-coating techniques rapidly evaporates the dispersion solvent, which can allow for maintaining the solution morphology (and potentially aggregation) from the ink dispersion [30]. Two common fabrication techniques are a catalyst-coated substrate (CCS)/gas diffusion electrode (GDE) (Figure 6A), where the CL is coated onto the gas diffusion layer (GDL), or a catalyst-coated membrane (CCM) (Figure 6B), where the CL is coated directly onto the AEM prior to cell assembly [127]. The CCS can allow for lower Pt loadings and large active areas, though it is more prone to AEM-CL surface resistances [20,128]. In contrast, the CCM is often found to have less contact resistance, likely due to the direct contact between the CL and membrane [20,127]; however, it is dependent on the AEM having good physical properties whilst being dry to withstand CL application [20].

Fabrication techniques were shown to play an important role in the performance of the aforementioned PIL BCP poly(MMA-*b*-MUBIm-HCO_3_) [117]. The techniques compared were painted GDL (catalyst ink hand-painted onto GDL), air-sprayed GDL (airbrush used to apply catalyst ink to GDL), and decal transfer (catalyst ink painted onto Teflon-coated decals and then transferred to AEM) with the MEA sandwiched during fuel cell assembly or heat-pressed (painted GDL and air-sprayed) [117]. It was found that the painted GDL provided the best performance (0.0293 W cm^−2^) compared to the air-sprayed (0.0154 W cm^−2^) and decal transfer (0.0076 W cm^−2^) GDLs [117]. The process required to perform the decal transfer involves a high temperature and pressure, which were suspected to have a negative impact on the CL [117]. In contrast, the painted and air-sprayed techniques were suspected to provide more triple-phase boundaries within the morphology, particularly with the painted GDL [117]. These results underscore the importance of selecting fabrication methods that preserve CL structure and maximize triple-phase boundary formation, with painted GDL emerging as the most effective technique for PIL-based AEIs. In a recent study, cathodes with varying PiperION^®^ AEI contents were prepared using inkjet printing [121]. This technique has been used in PEMFCs for precise CL fabrication; however, the application to AEMFC is first reported by Liu and Secanell [121]. Additional method options more often seen with other fuel cell applications include blade coating [129,130] and screen printing [131]. A comparative table of fabrication methods is presented below in Table 7 to highlight their differences in process as well as important aspects to consider prior to method selection and implementation.

Studies on AEI content optimization have found that increasing the amount of AEI in the catalyst ink aids in ion transport channel formation within the CL [28,87,121]. However, when in excess or when using a suboptimal ink formulation, the AEI can form aggregates that block pathways for reactant gases [28,121]. The aggregation of AEIs is a result of weak interaction with carbon, which creates a non-uniform distribution that limits performance [28]. Much like other characteristics of the MEA, the AEI quantity appears to vary with the selected ionomer material and fabrication parameters; therefore, individual optimization is required when assessing different AEIs [132]. The effect of the AEI content is not as widely studied as other aspects of CL fabrication [87,133]. The optimization of ink dispersion formulas, particularly with ionomer alterations [87,122], is important to improving AEMFC performance [87]. Recent developments providing insight for overcoming AEMFC challenges through optimizing ink dispersions in addition to operating conditions will be presented in the following section. As there are not currently commercial standards for AEMFC testing [21], the selected control comparisons for studies can vary between previous studies and the currently commercially available ionomers.

### 3.1. Impacts of Alterating Parameters on Performance with Fumion^®^

Although the most commonly used catalyst in current fuel cell studies is Pt [20], an alkaline environment can decrease activity, and as such, the compatibility between the AEI and Pt is of high importance to ensure high AEMFC performance [84]. Current commercially available ionomers, such as Fumion^®^, have been used for the purpose of assessing AEI–catalyst compatibility [20,30,122,123]. Chae et al., Saidin et al., and Han et al. have recently conducted studies using a Fumion^®^-type polymer (FAA-3-SOLUT-10) as the AEI to assess the impact of altering fabrication parameters on performance [20,30,122], as summarized in Table 8. Chae et al. investigated the use of CCM versus CCS along with alterations to the AEI/catalyst ratio (0.5–1.5) in the electrodes, using a Fumion^®^ membrane (FAA-3-30) and Pt/C or PtRu/C (60 wt%) [20]. CCMs showed that the increasing AEI/catalyst ratio (up to 1.5) increased the agglomerate size while decreasing thickness and porosity [20]. Decreasing the CCM AEI/catalyst ratio (<1) decreased the agglomerate and pore size, resulting in a thicker and more porous CL [20]. Thickness changes are important to consider, as Fumion^®^ has previously shown that the ORR performance and ionomer catalyst interface are influenced by the AEI film thickness in RDE tests conducted by Liu and coworkers [123]. For a CCS, increasing theAEI content aided in ionic channel formation between the AEM and CL; however, aggregation and porosity loss caused high charge transfer and mass transport resistances [20]. The CCMs showed improved performance as the ohmic resistance was lower (~0.1 Ω cm^2^) compared to the CCSs, likely due to the direct CL to AEM contact [20]. An AEI/catalyst ratio of 1 was the best PPD for each method in single-cell tests, as it allowed the CCM to form ionic pathways without limiting the ECSA and for the CCS to decrease ohmic resistance [20]. The overall mass transport when using CCS is lower versus CCM due to the influence of the AEI content on morphology and performance; thus, high compatibility between the AEI and AEM is a necessity with this fabrication method [20].

Similarly, Saidin et al. varied the FAA-3-SOLUT-10/Pt/C (AEI/catalyst) ratio by mass from 20–60 wt% AEI content in the electrodes using the CCS method and a Fumasep FAA-3-PK-75 AEM to determine the optimum combination for performance [122]. Analysis of the fabricated CL surface structures showed that the pore size decreased with increasing ionomer content [122]. Similarly to Chae et al. [20], it was determined that 50 wt% AEI (i.e., AEI/catalyst ratio ~1) demonstrated the highest performance in this study (PPD 0.067 W cm^−2^) owing to uniformity [122]. This was attributed to the moderate AEI quantity and distribution improving the catalyst utilization without blocking transport pathways, and exceeding this amount resulted in more aggregation [122]. Increasing the AEI content to 60 wt% decreased performance and increased resistances, likely due to the AEI blocking transport pathways [122]. Through fuel cell tests, it was concluded that <50 wt% was likely too low an AEI content to create adequate ion-conducting channels, resulting in poor performance [122]. This is similar to the findings in Sebastián et al., where using Fumion^®^ AEIs, a Pt catalyst, and the CCS method had an optimal AEI content of 50 wt% of the electrode, while a lower content (25 wt%) was insufficient to cover the catalyst and greater (>58 wt%) caused performance decays attributed to Fumion^®^ blocking catalyst pores [132]. This is consistent with the results found in Chae et al., where an even (1:1) distribution of FAA-3-SOLUT-10 ionomer to catalyst produced optimal performance [20]. However, the PPD found in this study was much lower than those presented in Chae et al., despite using similar materials, indicating there are more aspects of fabrication methods beyond the ionomer content that should be investigated to achieve high performance.

**Figure 8:**
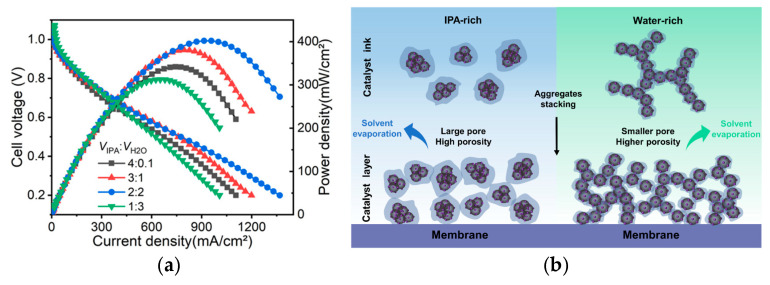
(**a**) CCM with varying IPA/water solvent ratio. Anode Pt loading: 0.4 mg cm^−2^, cathode PT loading: 0.5 mg cm^−2^, cell temperature: 60 °C, 100% RH, from Han et al. [30]. Reprinted (adapted) with permissions [30]. Copyright © 2024, American Chemical Society (**b**). Graphical illustration of solvent effects on the CL morphology, from Han et al. [30]. Reprinted (adapted) with permissions [30]. Copyright © 2024, American Chemical Society.

In contrast, Han and coworkers investigated the influence of solvent modifications to the cathodic CL morphology when using Fumion^®^ FAA-3-SOLUT-10 ionomer with a constant AEI/catalyst ratio (by mass) of 0.6 in both electrodes on CCMs with Fumion^®^ FAA-3-20 membranes [30]. For the cathode preparations, the volumetric ratio of IPA–water was varied between 4:0.1, 3:1, 2:2, 1:3, and 0:4 [30]. The anode volumetric ratio of IPA–water was kept constant at 11.5:1, and a Pt/C (70 wt%) catalyst was utilized to prevent flooding by limiting the thickness [30]. The solvent ratios with higher IPA contents resulted in larger aggregates forming more voids, while networks of the connected AEI/catalyst formed when the IPA–water ratio was less than 3:1 [30]. Further changes from increasing both the IPA and water contents (2:2) resulted in a water-rich environment that produced a 3D network of primary aggregates of ionomer covering the Pt/C and secondary aggregates interconnecting primaries [30]. The authors concluded that a higher IPA content (4:0.1 and 3:1, or greater) increases aggregation and inhibits dissociation of the QA due to low-energy solvent–ionomer interfaces [30]. It was also concluded that a higher water content results in better coverage of AEI on the catalyst, stronger QA dissociation, and 3D network branches due to higher carbon–solvent surface tension (illustration in Figure 7b) [30]. This was reflected in the AEMFC performance curves (Figure 8a), where the PPD increased as the solvent ratio switched from an IPA-rich to an equal IPA–water dispersion (4:0.1 up to 2:2) [30]. Further increasing the water content to an IPA–water ratio of 1:3 had a negative impact on the ECSA and the membrane during CCM fabrication [30]. It was concluded that the cathode CLs have a stronger 3D network with more Pt utilization, porosity, and ionomer catalyst distribution with the balanced (2:2) solvent ratio [30]. While the performance improvements were found within the context of utilizing Fumion^®^, some of the highest reported PPDs for AEMFC tests are 3.4 W cm^−2^ (3400 mW cm^−2^) in Huang et al. [58,134] (using ETFE-*graft*-PS-based ionomers) and 1.6–3.2 W cm^−2^ [36] in Ul Hassan et al. (using norbornene-based ionomers), which are significantly higher than the PPDs of Fumion^®^ in the presented studies. This is an example of the impact of ionomer selection and development on overall performance. The alterations to fabrications methods presented so far indicate the potential for AEMFC improvement that can be further combined in future optimizations of higher-performing ionomers.

### 3.2. Influence of Backbone Conformational Changes on Catalyst Binding

In AEI development, the influence of conformational changes on overall AEI performance has been discussed. This influence extends further into how conformational changes can alter and improve the fabricated CL. Zheng et al. compared poly(N-methyl-piperidine-co-m-terphenyl) (*meta*-QAPPT) [23] to poly(N-methyl-piperidine-co-p-terphenyl) (*para*-QAPPT) (previously synthesized and studied by Peng et al. [135]). This study assessed the influence of a small structural change (*meta*- vs. *para*-terphenyl) in the ionomer on CL structure as the temperature and humidity conditions changed [23], based on previous findings that the AEI structure and physical properties can influence the MEA performance [23,36,58,126]. Consistent with previous studies, the introduction of ionomer to ink dispersions resulted in aggregates from the AEI–catalyst binding [23]. Comparing ink dispersions of the two ionomers, the *para*-QAPPT had a higher SR (10.98%), which led to a wider size distribution of aggregates and higher viscosity than the *meta*-QAPPT with a smaller SR (4.21%) [23]. The differences in aggregation can influence the morphology of the AEI–catalyst interface within the CL [23], as the ink dispersion morphology can transfer to the final sprayed CL [30]. Zheng et al. fabricated CCMs on a PiperIon^®^ AEM with *meta*-QAPPT and *para*-QAPPT using the same preparation conditions for both AEIs [23]. Using *meta*-QAPPT produced a dense and uniform CL of 10.88 mm thickness, while *para*-QAPPT produced a thicker (11.40 mm) CL with a higher porosity and quantity of aggregates [23]. Molecular dynamics simulations indicated that the *meta*-QAPPT had the ability to fold in a more compact and dense structure in the CL (higher conformational density), due to its smaller volumetric increase ratio (i.e., less swelling) [23]. Through adsorption kinetic tests with varying RH (0–30%) and temperature (25 °C and 80 °C), the authors found that the *para*-QAPPT CL had higher water diffusivity, likely due to its higher porosity [23]. At high temperature (80 °C) and low RH (0–30%RH), the *meta*-QAPPT CL was reported to retain water due to its lower water diffusivity [23]. The authors expected the properties of the *meta*-QAPPT to improve the ionomer–catalyst interface and CL stability throughout cycling or humidity changes, which was shown in its performance compared to the *para*-QAPPT [23]. Imaging of the CL surface structures showed that *meta*-QAPPT produces homogeneity, while the aggregation with *para*-QAPPT produces cracks that can hinder continuity of ion conduction and water and gas distribution [23]. A meta conformation has been found to improve ionic channels in AEIs [102], which can be connected to how the *meta*-QAPPT in this study allowed for a uniform and dimensionally stable CL for AEMFC operations [23].

**Figure 9:**
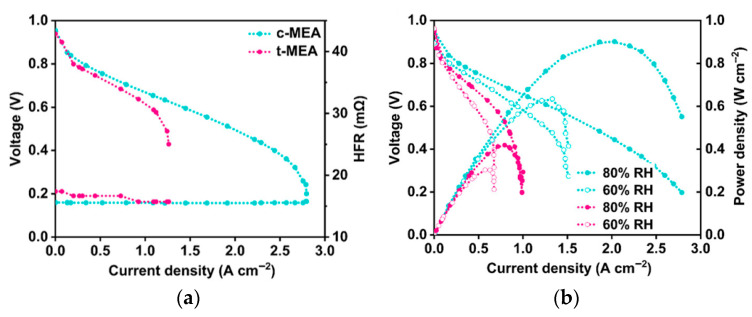
(**a**) Polarization curves and the HFR as a function of current density at 70 °C and at 100% RH. Reprinted (adapted) with permissions [136]. Copyright © 2023, American Chemical Society. (**b**) Polarization curves and power density curves at different RH (70 °C). Reprinted (adapted) with permissions [136], Copyright © 2023, American Chemical Society.

The performance difference between cis and trans isomers in the backbone of a stilbene (SB) synthesized with diphenylethane (DB) AEI to create poly(*cis*- or *trans*-stilbene-co-diphenyl piperidinium (SB-DB)) was compared in Yu et al. [136]. The ionomers were both found to be thermally stable and have good tensile strength (29 MPa for *cis*- and 37 MPa for *trans*-) [136]. The *cis*-ionomer was found to have a non-planar structure that prevented phenyl adsorption effects while also containing small free volume cavities for higher oxygen transport and water adsorption in comparison to the more planar *trans*-ionomer with less free volume [136]. Density functional theory (DFT) was used to determine that the *cis*-ionomer has only one phenyl ring parallel to the Pt catalyst, which prevented phenyl adsorption on the catalyst unlike the *trans*-ionomer [136]. The low adsorption additionally allowed for a more uniformity in *cis*-ionomer distribution as a binder in the CL [136]. In contrast, the *trans*-ionomer would heavily bind to partial particles of the catalyst, while other particles were not ionomer-bound [136]. The oxygen permeability and ECSA were found to be significantly higher with the *cis*-ionomer [136]. AEMFC testing (Figure 8a) further showed the property improvements, as the *cis*-ionomer had a PPD of 1 W cm^−2^ while the *trans*-ionomer was at 0.64 W cm^−2^ [136]. Humidity changes indicated that the MEA with the *trans*-ionomer was more humidity-dependent, as RH decreases had a more significant effect on PPD than with the *cis*-ionomer (Figure 9b) [136]. Similarly to the *meta*-QAPPT and *para*-QAPPT of Zheng et al. [23], a conformational change in the polymer structure seen with the *cis*-SB-DB and *trans*-SB-DB had a significant impact on performance due to polymer behavior within the CL [136]. Backbone conformational changes were shown in these cases to influence the polymer folding. As with further polymer development, this can be incorporated as a technique to improve ionomer binding to the catalyst for optimal CL fabrication.

### 3.3. Polymer Development and Modifications for Optimizing the Catalyst Layer

The use of newly developed ionomers is being further investigated to optimize AEMFC performance within the CL. Fraser et al. developed the ionomer DMP-PHPI-M, which contains poly(imidazolium) quaternary ammonium groups, for improving HOR/HER activity [84]. Their study found that the AEI structure allowed for the use of a higher ionomer content and provided a higher CL stability compared to AEI-free CLs [84]. The AEI/catalyst ratio was varied from 0 to 0.196, and it was found that the ECSA had slight decreases when above a ratio of 0.1 [84]. The authors speculated that reactions such as the HOR and Pt phenyl oxidation reactions decreased the pH and affected the ECSA [84]. As previously mentioned, a frequent challenge with the design of phenyl-containing polymers is associated oxidations with the Pt catalyst [94,126,137,138]. Fraser et al. concluded that the morphology of the CL at the AEI–carbon binding site caused cluster formations, wherein the oxidation only affected Pt near the outer cluster AEIs, while secondary pores allow for inaccessible Pt to be unaffected [84]. The analysis of the polymer in this study did not extend to AEMFC single-cell tests, which would be an area to explore further in terms of CL optimization.

**Figure 10:**
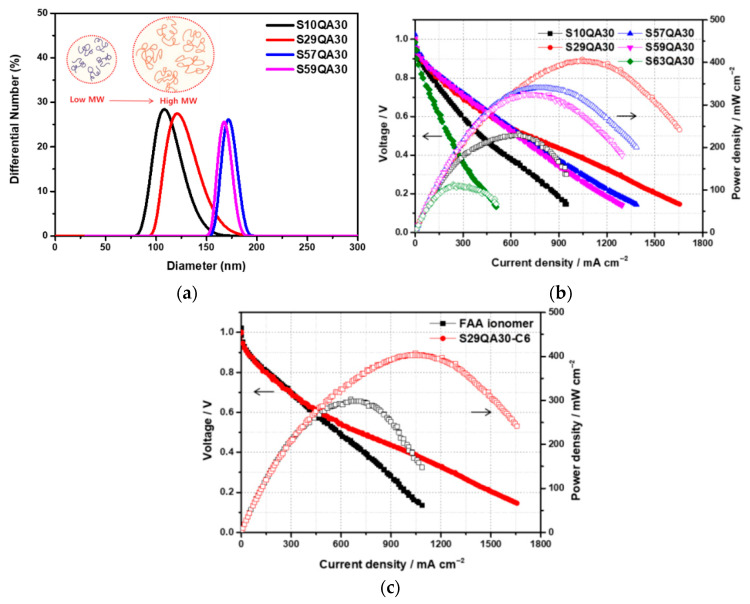
(**a**) Dynamic light scattering analysis of SxxQA30 ionomers to display molecular weight. Adapted from Chae et al. [80]. Copyright © 2021, the authors (licensed under: CC-BY 4.0). (**b**) AEMFC performance due to molecular weight effects on SxxQA30 AEIs with 0.4 mg cm^−2^ Pt on anode/cathode, 100%RH, no backpressure, and 60 °C. Adapted from Chae et al. [80]. Copyright © 2021, the authors [80] (licensed under: CC-BY 4.0). (**c**) AEMFC performance of S29QA30-C6 compared to Fumion^®^ (FAA-3-Solution) operating with Fumasep FAA-3-20 AEM, 0.4 mg cm^−2^ Pt on anode/cathode, 100%RH, no backpressure, and 60 °C. Adapted from Chae et al. [80]. Copyright © 2021, the authors [80] (licensed under: CC-BY 4.0).

In the previous Section 2.2.6, molecular weight changes caused by varying the styrene ratio in SxxQAyy-Cn (Figure 10a) made an impact on the CL morphology [80]. The higher molecular weights led to a tendency to aggregate within the ink dispersions, while the lower molecular weights had weaker binding to the catalyst [80]. In Figure 10b, it is shown that those improvements due to the impact of the molecular weight and quantity of styrene on the CL are reflected in the AEMFC performance [80]. This extended to the best-performing AEI (S29QA30-C6) outperforming Fumion^®^ (FAA-3-solution) (Figure 10c) [80]. This example shows how the improvements to the AEI properties themselves, such as targeting moderate molecular weight, can have an impact on the improvement of the CL fabrication.

Incorporation of fluorine-containing groups has often been used as a method to improve ionomer properties and performance [35,48,49,51,53,54,125,139]. In Yu et al., the effect of fluorination for improving the AEI and the triple-phase boundary was assessed by comparing a phenyl backbone substituted with a methyl group (CH_3_) versus substituting with a trifluoromethyl (CF_3_) group [125]. Comparisons of the morphology as an AEM and ^−^OH conductivity illustrated how the hydrophobicity of the CF_3_ group aided in hydrophobic/hydrophilic phase separation and improved conductivity [125]. The authors predicted that as a catalyst binder, the low polarizability of the fluorine groups in the CF_3_ ionomer would improve oxygen transport within the triple-phase boundary, which was validated using RDE tests, as it had a higher limiting current density compared to the CH_3_ ionomer [125]. Molecular dynamics simulations determined that oxygen becomes evenly distributed in the CL when using CF_3_, while in contrast, oxygen assembles in groups at the surface when using CH_3_ [125]. CCMs were prepared under the same conditions for each ionomer with a Pt (cathode) or PtRu (anode) catalyst loading of 0.5 mg cm^−2^ [125]. AEMFC testing under the same conditions resulted in a PPD for the CF_3_ ionomer of 1.2 W cm^−2^ without backpressure and 1.7 W cm^−2^ with 0.1 MPa, which was significantly higher than the CH_3_ ionomer values of 0.7 W cm^−2^ and 1 W cm^−2^ at the same respective backpressures [125]. Analysis of oxygen transport resistances showed that the CF_3_ ionomer had slightly lower ohmic resistance than the CH_3_ ionomer (41 mΩ cm^−2^ vs. 49 mΩ cm^−2^, respectively, at 100%RH). In a 100 h durability test under 0.4 A cm^−2^, the CF_3_ and CH_3_ ionomers had voltage decline rates of 1.8 mV h^−1^ and 2.4 mV h^−1^, respectively, further indicating the AEIs’ influence on overall AEMFC performance [125]. This study outlines that a method of small ionomer design modifications (CH_3_ to CF_3_) for improved morphology, mechanical properties, durability, and conductivity can have a major impact on the CL and overall AEMFC performance [125]. A comparison of the results when making the above-mentioned modifications are provided in Table 9.

### 3.4. Influence of Ink Dispersion Solvent on CL Morphology

As found with Fumion^®^, the influence of aggregation in CL ink dispersions on morphology and performance was of interest in Hyun et al. when studying solvent effects with *meta*-poly(terphenylene) (*meta*-TPN1) [103]. This study utilized organic solvent (DMSO, MeOH, IPA) changes in the ink dispersion to modify aggregation, with a 7:3 ratio of organic solvent (DMSO, MeOH, IPA) to water [103]. The authors predicted that the large polarity difference between the *meta*-TPN1 backbone and tethered ionic group would have different solvent interactions, thus varying the aggregation between solvents [103]. The dispersion and intermolecular forces had a significant influence on *meta*-TPN1, resulting in varying hydrodynamic diameters, with DMSO producing the smallest [103]. Assessment of the solvation states indicated that solvent systems with low solvation (IPA/water) resulted in the incomplete dissociation of the ion (Br^−^) and the trimethyl ammonium (TMA) in the ionic component, thus forming aggregations, while full dissociation was achieved in DMSO and MeOH systems [103]. These results indicate how the ideal solvent for morphology can be ionomer-dependent [103], as previous examples showed success when simply altering the quantity of IPA and water in the dispersions [30]. The morphology translated to the CL using the DMSO, MeOH, and IPA systems, as the DMSO had the highest thickness and uniformity, MeOH had moderate thickness and was highly porous, while IPA had a dense structure (aggregates) with low distribution [103]. The AEMFC performance was assessed based on comparison of one electrode at a time (i.e., anode constant/cathode varied, anode varied/cathode constant) where the DMSO CL was used as the constant or control [103]. The analysis of changing the cathode showed that the ionomer distribution of the DMSO CL improved ^−^OH conductivity and ECSA as the uniformity increased the triple-phase boundary [103]. The charge transfer resistances corresponded to the improved triple-phase boundary, as there was low resistance with DMSO and high with IPA [103]. In terms of the cathodic CL, the solvent changes highlighted the importance of creating a uniform ionomer distribution for performance in the water-deficient electrode [103]. In terms of the anodic CL, the MeOH CL had the best cell performance, likely attributed to the benefits of high porosity in the water-rich electrode, as it is more limited by H_2_ transport than the cathode [103]. The use of backpressure in both electrodes or only the anode indicated a decrease in mass transfer resistances within the anode, which indicated a relationship between the two electrodes that could be further investigated [103]. This study focused on alterations to aggregate size in ionomer dispersions through catering the solvent to ionomer needs, highlighted the complexity of the CL, and it offered an additional pathway to modify electrode fabrication [103].

In another study, Hyun and coworkers decreased the aggregate size in ink dispersions and unclogged pores of m-TPN1 CLs using a pyrene carboxylic acid (PCA) coatings on the carbon support of Pt catalysts [28]. The PCA was used for its carbon surface modification by π-π bonds formed with carbon and coulombic interactions with the QA of the AEI [28]. The DMSO/water solvent was used as it improved uniformity in a previous study, and PCA was added to only the cathode CL dispersion (no PCA in the anode) [28,103]. The PCA was used as an intermediate to anchor the AEI onto the catalyst particles for a uniform distribution [28]. Analysis of the CL morphology with varied PCA contents (10, 20, 35 wt%) showed that the size of aggregations decreased with the increase in PCA, displaying uniformity and more ideal pore distributions at both 20 and 35 wt% [28]. Adhesion to the AEI was stronger with PCA (45.4 kcal mol^−1^) than without (11.2 kcal mol^−1^) when compared to the untreated carbon control [28]. AEMFC performance improved with the addition of PCA, the highest-performing being the PCA content of 20 wt%, which had a smaller Pt particle size, reduced resistances (mass transfer and kinetic), and a larger ECSA compared to the 35 wt% [28]. The improved morphology found by the authors indicated not only the impact of the CL ionomer distribution but also the benefits of an intermediate coating such as PCA to AEMFC performance [28].

## 4. Current Progress in Water Management for Improved Performance

**Figure 11:**
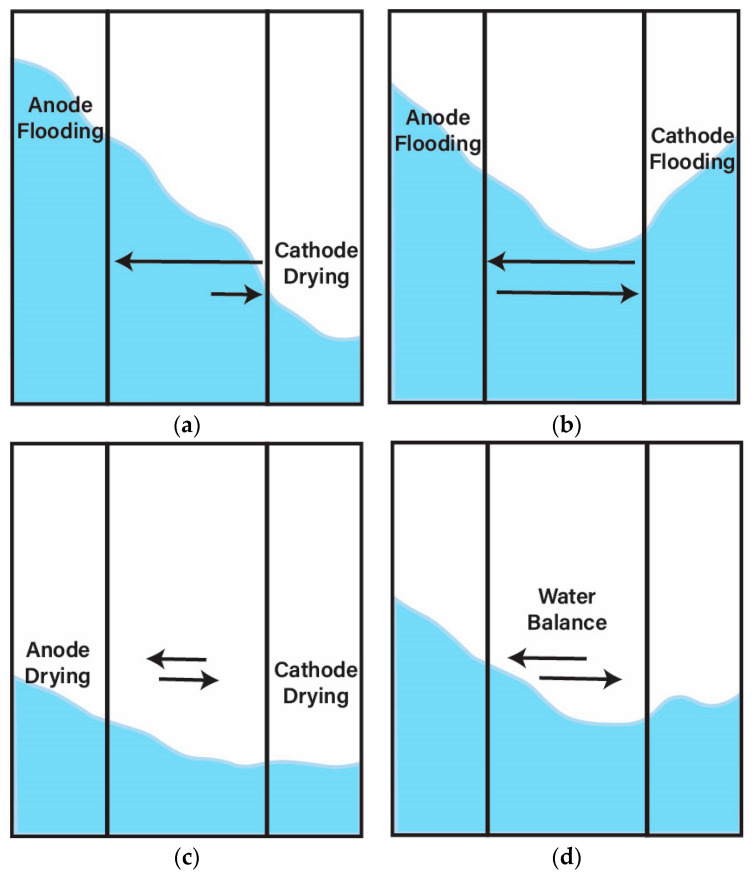
Illustration of potential water management scenarios, where Arrows indicate the direction and relative magnitude of water transport across the membrane. (**a**) Water accumulation at the anode and away from cathode. (**b**) High water content resulting in flooding throughout. (**c**) Too low a water content resulting in drying of both electrodes. (**d**) Decreased anode flooding and maintained hydration at cathode through water management strategies.

Performance losses over time remain a major concern in AEMFCs, prompting efforts to optimize water management and address other factors that hinder long-term stability. Hydration of the cell is required for proper AEM and AEI function; however, excess water can result in flooding, as shown by the water management scenarios illustrated in Figure 11 [34]. Dehydration of the cell during AEMFC operation has been predicted to cause degradation and an increase in HFR over time, especially when operating at higher temperatures [121]. The advancement of laboratory-scale studies is needed to develop the understanding of the connection between water management, the CL, and the triple-phase boundary [87] prior to reaching industrial-scale needs. Water management strategies in terms of the technology components focus on WU in the AEM and AEI, hydrophobic treatment of the GDL (PTFE), preparation methods of the CL, inlet gas humidity, and gas flowrates [34,140]. Previously discussed ionomer developments and investigations into CL interactions can highlight these strategies. In previous water management studies, the focus tended to be on the AEM while the AEI was overlooked. Along with an AEM of good performance, the selected AEIs are required to aid in overcoming the flooding conditions at the anode and the drying conditions at the cathode for achieving optimal AEMFC performance [108]. Utilizing AEIs with different IECs (particularly with a high IEC) has been found to benefit water management between the anode/cathode and improve performance [35,36,118,141]. The AEI property modifications can be used in tandem with optimizing the operating conditions and fuel cell configuration to further improve performances.

A deeper understanding of water management throughout the MEA can be explored using modelling and quantification techniques. A three-dimensional model of the anode was presented by Huo et al. for analyzing material transport (i.e., liquid water and gas) through the GDL and CL [33]. The anode CL liquid water volume fraction showed a linear relationship in response to increasing inlet gas RH steadily up to 70% RH, a sudden steep increase between 70–80% RH, and constant volume fraction (plateau) above 80% RH [33]. The quantity of liquid water in both the GDL and CL decreased linearly with the increase in porosity [33]. Increasing the current density from 0.05 to 1.45 A cm^−2^ resulted in a fraction of liquid water increases in the GDL (0.096 to 0.260) and in the CL (0.088 to 0.236) [33]. The increase in current density at the anode creates a larger difference between the water concentration at the anode and cathode, resulting in water diffusion from anode to cathode [33]. Huang et al. used quantification methods to investigate water management within the anode (0.6 mg_PtRu_ cm^−2^) and cathode (0.4 mg_Pt_ cm^−2^) with a PiperION^®^-A5 AEI and AEM [142]. Electrochemical impedance spectroscopy (EIS) and distribution of relaxation time (DRT) were applied to transform the resulting Nyquist plot into a spectrum of resistivity versus frequency [142]. Integration of the DRT peaks corresponding to the impedance of each polarizations allowed for performance losses to be quantified [142]. Increasing current density caused the kinetics-limiting factor to change from ion to mass transfer resistance [142]. Flooding at the anode reduced ion transport resistance (by 37.1%) and increased charge transfer and mass transport resistance (by 61.8% and 219.2%, respectively), while the cathode had a lower increase in charge transfer resistance (by 33.5%) and did not affect the mass transport resistance significantly [142]. Ion transport resistance contributed to the performance declines the most under cathode-drying conditions (A/C %RH 90/70), causing a more rapid decline (0.3 V drop after 23 h) than anode-flooding conditions (A/C %RH 110/90, 2.03 mV h^−1^ degradation rate in first 40 h) [142]. The degradation of the anode in normal conditions (A/C %RH 90/90) was higher at 4.35 mV h^−1^ [142]. The connection of anode CL pore structures and water management was reported in Xiao et al. and included both AEMFC tests and phase modelling [143]. The AEM and AEI in this study were both PiperION^®^-A5, while the anode CL had a varied carbon powder content to further investigate porosity influence [143]. The polarization curves were delineated using the method reported by Gasteiger et al. [144] to determine the kinetic, ohmic, and mass transfer limitations [143]. Increasing the carbon in the CL allowed for a PPD increase (1.56 W cm^−2^ to 2.10 W cm^−2^), and improved limiting current density (4.10 A cm^−2^ to 5.20 A cm^−2^) [143]. This was attributed to the improvements in pore distribution and volume enhancing water management [143]. A phase field model was used to assess the porosity impact, and it was found that having a gradient porosity created a capillary pressure that enhanced water transport [143]. Sarker et al. utilized neutron radiography and modelling to study water transport [145]. This study used a sulfonamide-linked alkyl ammonium perfluorinated Gen 2 polymer seen in Park et al. [146] for the AEI and AEM [145]. Sarker et al. used an analytical model using Python that accounted for different aspects of the AEMFC, such as the electrochemical reaction kinetics, and the transport of water, mass, electrons, and ions [145]. In addition to the type of ionomer preparation for GDEs, the sensitivity of RH changes, H_2_, and O_2_ were also analyzed [145]. A comparison of AEI preparation methods indicated that AEMFC performance was sensitive to samples that did not fully anion exchange to hydroxide form during preparations [145]. Modelling of higher flooding conditions found that anode flooding at high current density results in a sharp performance decline [145]. The modelling of asymmetric RH conditions indicated that decreasing the cathode RH results in back-diffusion of water from anode to cathode and reduces electroosmotic drag of water from cathode to anode [145]. This model indicated that a reduction in cathode RH is a viable method to prevent anode flooding [145], which is a water management method discussed later on in this section. The concentration of anodic H_2_ was identified as the main limiting factor for current density due to the large quantity of water at the anode [145]. Understanding the behavior of water distribution and the effect of AEMFC operation on the MEA can aid in advancing AEI design strategies.

Modifications to the IEC for altering water uptake properties have been used as a part of water management strategies; however, it can have an impact on other aspects of the AEI performance, such as gas transport. Lu et al. reported the effects of IEC modifications when utilizing methylpiperidinium-functionalized polyethylene (PEPM) AEIs (IECs 2.25 and 2.07) [39]. Cyclic voltammetry (CV) and rotating disc electrode (RDE) tests were performed using neutral and positive N,N,N′,N′-tetramethyl-*p*-phenylenediamine (TMPD) and negative 1,4-benzoquinone (BQ) to analyze the transport properties of the AEIs [39]. The electrostatic repulsion of the cationic AEIs made TMPD^+^ production irreversible, with this effect increasing with IEC [39]. In contrast, the BQ^−^ ions were concentrated within the ionomer, causing the large anodic peak currents [39]. Gas permeability coefficients determined using neutral TMPD that the permeability decreased with the PEPM at IEC 2.25 compared to the IEC of 2.07 [39]. This indicated that the increase in cationic groups associated with the IEC increase might create barriers that obstruct the transport of neutral species [39]. This is an important consideration, as this can indicate obstruction of neutral reactant gases (i.e., O_2_ and H_2_). Additionally, the AEMFC performance was analyzed using symmetric and asymmetric (A/C IEC: 2.07/2.25) electrode configurations [39]. The fabrication of the CL indicated that the higher IEC (2.25) was better suited for the n-propanol solvent than the low IEC (2.07) and was better dispersed in the ink solution [39]. The better dispersion translated into the AEMFC tests, as the IEC 2.25 in the symmetric configuration had the best performance (~350 mW cm^−1^, 80 °C) compared to the IEC 2.07 (~165 mW cm^−1^, 60 °C) and the asymmetric configuration (~185 mW cm^−1^, 60 °C) [39]. The flooding effects were significant with IEC 2.07, and the temperatures were reduced from 80 °C to 60 °C to accommodate [39]. The analysis of these AEIs indicates important considerations of the impact IEC has within the CL [39].

In 2020, the first use of asymmetry in AEMFC configurations for water management was reported by Ul Hassan et al. [36] and Leonard et al. [35]. Ul Hassan et al. utilized polynorbornene-based tetrablock copolymers (GT32, GT64, and GT78) with varying IECs (1.88, 3.37, and 3.74, respectively) as the AEM and AEIs [36]. The study found that an AEI configuration with a high IEC/hydrophilic anode (GT78) and low IEC/hydrophobic cathode (GT32) with approximately 72/78% RH (anode/cathode) delivered the highest performance (PPD 3.2 W cm^−2^) [36]. The single-cell assembly included PTFE-treated GDEs, which were expected to aid in maintaining the water content between the AEM and cathode to prevent drying and degradation [36]. This asymmetric configuration was able to maintain 2000 h of stability under 0.6 mA cm^−2^, 0.3 L min^−1^ reactant gas flowrate, and minor humidity adjustments throughout, which produced a ‘benchmark’ of high performance for the field [36]. The configuration there differed from Leonard et al., where the opposite AEI set up was found to be optimal [35,36]. The authors attributed the opposite results to the higher water transport of polynorbornene AEM and AEIs compared to those utilized in Leonard et al. (FLN-based AEIs and hexyltrimethyl ammonium-functionalized poly(phenylene) (HTMA) AEM) [35,36]. Leonard and coworkers compared the performance of various symmetric and asymmetric configurations using the high IEC AEI, FLN-100 (3.5 mmol g ^−1^), and low IEC AEI, FLN-55 (2.5 mmol g^−1^), with high RH (100%) and low RH (50%) [35]. Of the configurations, the top-performing utilized the anode/cathode AEI configuration of FLN-55/FLN-100 and 100/50% RH [35]. This was suspected to offer the most balanced back-diffusion that prevented overaccumulation of water at the anode and bring in enough water to maintain cathode hydration [35]. The long-term stability test with the asymmetric FLN configuration showed that with replenishments of water to combat sources of voltage loss, the operation time could reach 933 h at 0.6 mA cm cm^−2^ [35]. These findings have led to further use of asymmetric configurations for optimizing water management and performance.

As previously mentioned, Shubair et al. synthesized a series of BCPs wherein a high-IEC (i.e., 3.40 mmol g^−1^) AEI was consistently utilized at the cathode and the AEI IEC was varied at the anode (i.e., 1.03, 2.70, 3.40 mmol g^−1^). Power density was more than doubled when the lowest-IEC AEI was employed at the anode, in agreement with the findings of Leonard and coworkers [118]. Hu et al. utilized a crosslinking strategy for improving the interactions of the AEM and the CL with propargyl-grafted PFBP-based polymers (x-Trip-PFBP-Pr-m) [147]. The crosslinking caused strong interactions between the AEI and AEM based on molecular dynamics simulations [147]. Similarly, the use of these ionomers produced a PPD greater than 1 W cm^−2^ when utilizing an asymmetric ionomer loading with a hydrophobic AEI (x-Trip-PFBP-Pr-30) at the cathode and a hydrophilic AEI (x-Trip-PFBP-Pr-10) at the cathode, allowing for 1000 h of stability [147].

**Figure 12:**
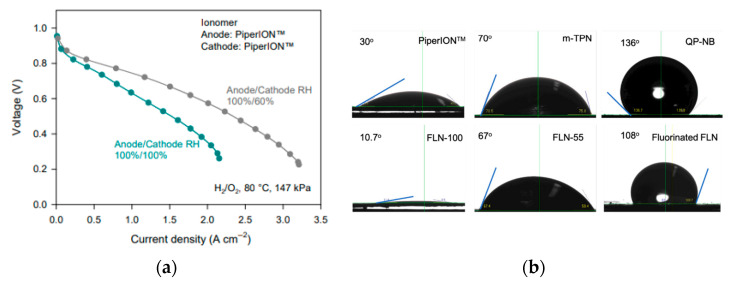
(**a**) AEMFC performance of PiperION^®^ with asymmetric and symmetric RH. Adapted from Leonard et al., Supplementary Information [24]. Copyright © 2022, the authors [24]. *Advanced Energy Materials*, published by Wiley-VCH GmbH (licensed under CC BY-NC-4.0). (**b**) Water contact angles of FLN, m-TPN, PiperION^®^, and QB-NB. Adapted from Leonard et al. [24], Supplementary Information [24]. Copyright © 2022, the authors [24]. *Advanced Energy Materials*, published by Wiley-VCH GmbH (licensed under: CC BY-NC-4.0).

In the study by Chen et al. on the performance of AEMFCs with poly(fluorenyl aryl piperidinium)-based polymers (PFBP), the RH for various tests was optimized based on performance, ultimately resulting in asymmetry of the anode/cathode RH (e.g., 75/100 in highest PPD) [54]. As reported by Leonard et al., a more hydrophilic AEI (e.g., FLN-100, PiperION^®^) can allow for a lower RH at that electrode site (Figure 12a), while AEIs with more hydrophobicity (e.g., QB-NB) can operate well at full humidification [24]. Although the use of AEIs with more hydrophilicity could lead to flooding and increased transport resistances from water accumulation, strong phase separation seen in polymers like FLNs decrease these effects from the inclusion of hydrophobic components [24]. The impact of the hydrophobic components on ionomer interactions with moisture can be seen through contact angle tests (Figure 12b) [24]. Chen et al. utilized asymmetry, finding that pairing water-permeable PFBP at the anode with high-WU PDTP-75 at the cathode performed well when 200 kPa backpressure and a PtRu anode catalyst was applied (2.08 W cm^−2^) [55]. The PDTP-type ionomers did not perform well at the anode, as the low water permeability and high WU likely had negative impacts on the water balance across the cell [55]. The MEAs were assessed after being almost stable at 0.4 A cm^−2^, 80 °C, and 100 h operation, and they did not appear to have clear degradation, which indicated that the durability was water-management-dependent [55]. Additionally, the highest performance (2.58 W cm^−2^) was found when pairing water-permeable PFBP AEIs with 75/100% anode/cathode RH [55].

In another study from Chen and coworkers on utilizing water management techniques through AEI selection and RH changes, the high WU and water permeability properties of PFBP-14 were suspected to decrease the anode flooding at low hydrogen flowrates [104]. However, the durability was limited to 100 h due to water management challenges, with ultimate anode flooding [104]. Further modifications with AEIs following asymmetric IEC loading in the electrodes could extend long-term durability to the level of the highest-performing AEMFCs of Ul Hassan et al. [36] and Leonard et al. [35]. As previously mentioned, the optimal asymmetric configuration of Ul Hassan et al. [36] (high-IEC/low-RH anode, low-IEC/high-RH cathode) was the opposite of the configuration found in Leonard et al. [35] (low-IEC/high-RH anode, high-IEC/low-RH cathode). Studies in asymmetric IEC and/or RH configurations [118,147] have tended to agree with Leonard et al. [35]. Alternatively, it was reported in Chen et al. [104] that the high water permeability of c-PAP AEMFCs required an anode/cathode configuration of 75/100% RH [54,55,104], which aligns more with the RH findings from Ul Hassan et al. [36]. However the asymmetric AEI configuration (water-permeable anode, high-water-uptake cathode) in Chen et al. [104] follows the Leonard et al. configuration [35].

Asymmetric electrode configurations by AEI IEC, hydrophobicity, or RH have consistently shown the ability to extend durability and improve power density when carefully tailored to the transport properties of each electrode. While some discrepancies remain across studies due to differing polymer chemistries and fuel cell architectures, a clear trend emerged: balancing hydration and preventing localized flooding or drying requires complementary AEIs and RH at the anode and cathode, rather than uniformity. Going forward, AEMFC researchers should integrate asymmetric design principles with optimization of electrode morphology, ionomer distribution, and GDL properties to maximize triple-phase boundary formation, sustain ion conductivity, and suppress degradation mechanisms. Ultimately, water management should be considered in every aspect of AEI design and cell engineering to meet the performance and durability demands of commercial AEMFCs.

## 5. Future Outlooks and Conclusions

The rapid expansion of AEI research has demonstrated that enhancing material properties alone is not sufficient to ensure high AEMFC performance. As presented in this review, improved performance arises from balancing polymer backbone and cation chemistry, morphology, electrode fabrication, and ionomer–catalyst interactions. Future AEI development should emphasize chemistries with proven stability and performance (e.g., PPO- and FLN-based backbones with piperidinium cations; ETFE-g-VBC with QA sidechains) while moving away from structures with recurring limitations (e.g., aryl-ether-based QA polymers such as Fumion^®^/FAA-3, which show poor alkaline stability; overly hydrophilic fluorene derivatives prone to excessive water uptake; imidazolium-functionalized ionomers with limited durability).

1. Molecular Design for Balanced Transport and Durability

AEI backbone chemistry and cation type must be optimized to balance ionic conductivity, water uptake, and chemical stability. Rigid aromatic backbones such as FLN, PPO, and PS offer superior alkaline stability, especially when combined with sterically hindered cations like piperidinium. However, these benefits depend on molecular weight being tuned to avoid catalyst layer (CL) agglomeration. Thus, medium-molecular-weight AEIs often perform best, enabling effective ion transport channels while minimizing ohmic losses. Among specific systems, ETFE-g-VBTMA-Cl ionomers [58,69,111,112,113] remain the highest-performing benchmark (>3 W cm^−2^), while aryl-ether QA systems such as Fumion consistently degrade under alkaline conditions and should no longer be relied upon.

2. Morphological Engineering via Polymer Architecture

The microstructure of AEIs within the CL is critical in device performance. Strategies such as block copolymerization and alkyl spacer incorporation increase ionic domain spacing, which promotes continuous ion transport channels; larger ionic domain spacing generally correlates with higher hydroxide conductivity. SAXS studies show that domains in the ~5–20 nm range are typically associated with higher hydroxide conductivity without loss of mechanical strength. Morphological control also allows for the decoupling of IEC and water uptake. While high water uptake (>100 wt%) can support strong conductivity, uncontrolled swelling (>50% expansion) leads to poor durability. For example, fluorene-based AEIs that absorb excessive water and lack dimensional stability fall into this category and should be avoided.

3. Electrode Configuration Tailored to AEI Function

AEI properties must be evaluated in the context of specific electrode roles. Asymmetric configurations, in which AEIs with different IECs or chemistries are used at the anode and cathode, have shown promise in regulating water distribution and optimizing local ionic environments. For example, pairing a high-IEC cathodic AEI with a low-IEC anodic counterpart can balance hydration and minimize flooding or dry-out. Results repeatedly show that this approach outperforms using identical AEIs on both electrodes.

4. Processing–Structure–Performance Integration

AEI effectiveness depends not only on material chemistry and morphology but also on distribution within the CL. Optimal performance requires that ionomers form continuous pathways to catalyst sites while maintaining porosity for gas transport and hydration. Achieving this relies on the interplay between AEI properties (e.g., solubility, viscosity, aggregation behavior) and electrode fabrication conditions (e.g., ink composition, deposition technique, drying dynamics). For example, solvent ratios in catalyst inks strongly influence ionomer dispersion and final CL morphology, impacting triple-phase boundary formation and ion transport. In practice, easily dispersed AEIs such as PPO and ETFE-g-VBC produce uniform electrodes and stable ionic networks. By contrast, poorly dispersing AEIs such as aryl-ether QA systems or linear/high-MW PS backbones can aggregate and reduce porosity. In these linear PS systems, phenyl adsorption onto Pt has been reported to cover catalyst sites and block gas access, whereas grafted ETFE-PS architectures avoid this problem and remain among the highest-performing AEIs.

5. Compatibility with Non-PGM Catalysts

AEI chemistry influences not only ionic transport but also catalyst dispersion and utilization, particularly for non-PGM catalysts where surface accessibility is critical. Functional groups such as imidazolium (e.g., in PILs and QILs) can improve ORR kinetics and dispersion, but their poor alkaline stability prevents long-term use unless stabilized by more robust backbones. Though this area is still in early stages of development, continued innovation in AEI–catalyst interactions will be essential for advancing cost-effective AEMFCs. Presently, most studies focus on optimizing AEI materials for high AEMFC performance with a Pt catalyst despite the desire to move towards non-PGM. It is unlikely that AEIs optimized for Pt will automatically transfer to Ni, Fe, or Cu catalysts. For example, in the field of anion exchange membrane water electrolysis (AEMWE), Aemion^®^ was found to be incompatible with Ni-based anodes [88]. There may not be a single “best AEI” for all catalysts, but ETFE-g-VBTMA-Cl- and PPO-based AEIs remain reliable choices for Pt systems, while more tailored solutions are needed for non-PGM.

Collectively, these key directions reinforce that no single variable can be used to determine AEI performance in isolation. Instead, future development must focus on an integrated design that pairs material synthesis with electrode fabrication. To reach commercial viability, AEIs must also be inexpensive, scalable, and compatible with large-scale processing (e.g., roll-to-roll). From a commercial perspective, the most common ionomers are Fumion^®^ (FAA-3), Aemion^®,^ PiperION^®^, and Sustainion^®^. Fumion^®^ is widely used but is not recommended as it suffers from poor alkaline stability and limited durability. Aemion^®^ provides higher chemical stability and conductivity than Fumion^®^, but it presents challenges in ink preparation and can swell excessively in the OH^−^ form, leading to blocked gas pathways. PiperION^®^ is a newer commercial option with promising stability, though less published performance data is available compared to Aemion^®^. Sustainion^®^ has also shown good initial performance, but imidazolium cations are chemically unstable in the long term in alkaline conditions. Overall, for researchers who prefer not to synthesize AEIs, Aemion^®^ currently offers the most practical commercial alternative to Fumion^®^, while PiperION^®^ may emerge as another option as more data becomes available. It would be highly valuable for future experimental studies to directly compare the leading commercial AEIs under consistent operating conditions. Such benchmarking would help clarify trade-offs in stability, conductivity, and electrode compatibility and provide researchers with clearer guidance when selecting commercial ionomers as alternatives to in-house synthesized materials.

## Figures and Tables

**Table 2:** Summary of AEMFC performances using piperidinium and/or poly(fluorenes) AEIs.

AEI ^a^	Performance	Ref.
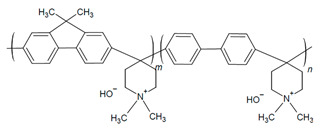 Poly(fluorene-co-biphenyl N,N′-dimethylpiperidinium) (PFBP)	IEC ^c^: 3.23 PPD: 2.34 W cm^−2^ AEMFC Conditions: 80 °C A/C Ionomer: PFBP-14/PFBP-14 A/C Catalyst (loading mg cm^−2^): PtRu (0.42)/Pt (0.33) Inlet A/C Fuel 1 L min^−1^/1 L min^−1^ 130 kPa backpressure 75/100% RH	[54] Also in: [55,104]
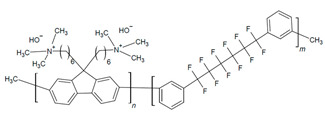 QPAF-4	IEC: 2.2 PPD: 0.226 W cm^−2^ AEMFC Conditions: 60 °C A/C Ionomer: QPAF4-C6-TMA/QPAF4-C6-TMA A/C Catalyst (loading mg cm^−2^): Pt (0.2)/Pt (0.2) Inlet A/C Fuel 0.1 L min^−1^/0.1 L min^−1^ No backpressure 100/100% RH	[51,105]
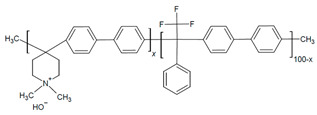 Poly(aryl piperidinium) based on biphenyl (PAP-BP-x)	IEC ^b,d^: 2.02 (x = 60), 2.38 (x = 70) PPD: 0.92 W cm^−2^ AEMFC Conditions: 95 °C A/C Ionomer: PAP-BP-100/PAP-BP-100 A/C Catalyst (loading mg cm^−2^): Pt (<0.15)/Ag-based (1) Inlet A/C Fuel Conditions: 0.15 L min^−1^/0.95 L min^−1^ 250/130 kPa backpressure 96/100% RH CO_2_-free air used instead of O_2_	[106]
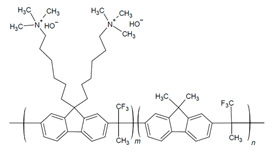 Quaternized poly(fluorenes) (FLN)	IEC ^c^: 2.5 (FLN55), 3.5 (FLN100) PPD: ~1 W cm^−2^ AEMFC Conditions: 80 °C A/C Ionomer: FLN-55/FLN-100 A/C Catalyst (loading mg cm^−2^): PtRu (0.75)/Pt (0.6) Inlet A/C Fuel Conditions: 1400/700 sccm 147.5 kPa backpressure 100/50% RH	[35] Also in: [48,49,63,94]

^a^ Structures drawn based on information provided in respective sources. ^b^ Obtained with Mohr titration. ^c^ Obtained via ^1^H NMR. ^d^ IEC of AEI used in AEMFC tests not reported.

**Table 3:** Summary of AEI performances reported comparing sidechains and spacers.

AEIs ^a^	Properties	Ref.
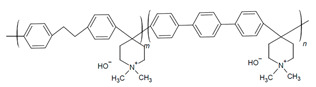 Poly(diphenylethane-co-terphenyl piperidinium) (PDTP)	IEC: 3.1 PPD: 2.08 W cm^−2^ AEMFC Conditions: 80 °C A/C Ionomer: PFBP-14/PDTP-75 A/C Catalyst (loading mg cm^−2^): PtRu(0.26)/Hispec Pt (0.26) Inlet A/C Fuel Conditions: 1 L min^−1^/1 L min^−1^ 200 kPa backpressure 75/100% RH	[55] Also in: [108]
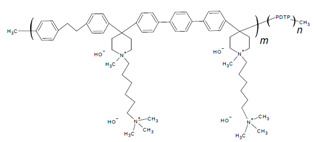 HTMA + PDTP (s-PDTP-x)	(For s-PDTP-65) WU: 178% (at 25 °C) SR: 54% (at 25 °C) IEC: 3.0 σ: 72 mS cm^−1^ (at 30 °C, ^−^OH form) PPD:1.47 W cm^−2^ AEMFC Conditions: 80 °C A/C Ionomer: s-PDTP-65/s-PDTP-65 A/C Catalyst (loading mg cm^−2^): Pt (0.5 or 0.6)/Pt (0.5 or 0.6) Inlet A/C Fuel Conditions: 1 L min^−1^/1 L min^−1^ 130 kPa backpressure 75/100% RH	[108]
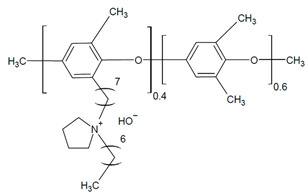 PPO-7Py7	IEC ^b^: 1.65 PPD: 0.261 W cm^−2^ AEMFC Conditions: 60 °C A/C Ionomer: PPO-7Py7/PPO-7Py7 A/C Catalyst (loading mg cm^−2^): Pt (0.5)/Pt (0.5) Inlet A/C Fuel Conditions: 0.5 L min^−1^/0.5 L min^−1^ No backpressure 100/100% RH	[25]

^a^ Structures drawn based on information provided in respective sources. ^b^ Obtained via Mohr titration.

**Table 4:** Summary of AEI performances reported for improved microphase separation and phenyl adsorption reductions.

AEI ^a^	Performance	Ref.
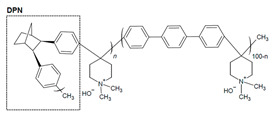 poly(norbornane-co-aryl piperidinium) (PDPN-n)	IEC ^c^: 2.54 PPD: 0.97 W cm^−2^ WU: 227.7% (at 30 °C, PDPN-26) SR: 61.8% (at 30 °C, PDPN-26) AEMFC Conditions: 80 °C A/C Ionomer: PDPN-26/PDPN-26 A/C Catalyst (loading mg cm^−2^): PtRu (0.5)/Pt (0.5) Inlet A/C Fuel Conditions: 0.4/0.4 L min^−1^ No backpressure ~85% RH (76 °C humidification tank)	[16]
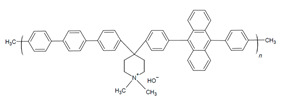 poly N-aryl piperidinium (PNAP-2)	IEC ^d^: 2.95 PPD: 2.07 W cm^−2^ WU: 73% (at 80 °C) SR: 30% (at 80 °C) ORR (Tafel): 65 mV dec^−1^ AEMFC Conditions: 80 °C A/C Ionomer: Synthesized PNAP ionomer A/C Catalyst: Pt (0.7 ± 0.1)/Pt (0.7 ± 0.1) Inlet A/C Fuel Conditions: 1 L min^−1^/1 L min^−1^ 200 kPa backpressure 75/100% RH	[75]
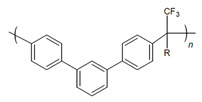 *Meta*-terphenyl (*meta*-TPN1)	IEC ^c^: 2.15 PPD: ~0.6 AEMFC Conditions: 80 °C A/C Ionomer: meta-TPN1/meta-TPN1 A/C Catalyst (loading mg cm^−2^): PtRu (0.2 ± 0.01)/Pt (0.2 ± 0.01) Inlet A/C Fuel Conditions: 1 L min^−1^/1 L min^−1^ No backpressure 100/100% RH	[103] Also in: [24,28,77,102,109]
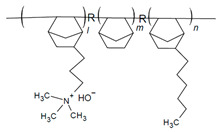 Quaternized polynorbornene(QP-NB)	IEC ^d^: 2.5 PPD: 1.41 W cm^−2^ AEMFC Conditions: 80 °C A/C Ionomer: QP-NB/QP-NB A/C Catalyst (loading mg cm^−2^): PtRu (0.5)/Pt (0.6) Inlet A/C Fuel Conditions: 1400/700 sccm 147 kPa backpressure 100/100% RH	[24] Also in: [36,57]

^a^ Structures drawn based on information provided in respective sources. ^c^ Obtained via back titration. ^d^ Obtained via ^1^H NMR.

**Table 5:** Summary of AEI performances reported using polystyrenes.

AEI ^a^	Properties	Ref.
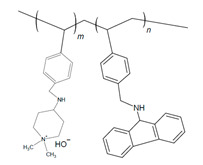 Amine piperidinium polystyrene (P-AP) m = 100, *n* = 0	IEC ^b^: 3.4 PPD: 1.35 W cm^−2^ WU: ~500% AEMFC Conditions: 80 °C A/C Ionomer: P-AP/P-AP A/C Catalyst (loading mg cm^−2^): PtRu (0.6 ± 0.1)/Co-Mn (1.2) Inlet A/C Fuel Conditions: 0.7/1 L min^−1^ 100 kPa backpressure 100/100% RH	[110]
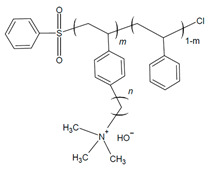 Functionalized bromoalkylated PS(SxxQAmm-Cn)	For S29QA30-C6: IEC ^c^: 1.47 PPD: 0.407 W cm^−2^ AEMFC Conditions: 60 °C A/C Ionomer: S29QA30-C6/S29QA30-C6 A/C Catalyst (loading mg cm^−2^): Pt (0.4)/Pt (0.4) Inlet A/C Fuel Conditions: 0.2 L min^−1^/0.4 L min^−1^ No backpressure 100/100% RH	[80]
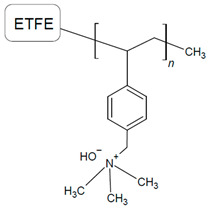 ETFE-g-poly(vinylbenzyltrimethylammonium chloride) [ET-FE-g-poly(VBTMAC)]	IEC ^b^: 1.24 ± 0.06 mmol g^−1^ PPD: 1.48 W cm^−2^ WU: 155.4 ± 1.8% AEMFC Conditions: 60 °C A/C Ionomer: ETFE-*g*-poly(VBTMAC)]/ETFE-*g*-poly(VBTMAC)] A/C Catalyst (loading mg cm^−2^): PtRu (0.8)/Pt (0.55) Inlet A/C Fuel Conditions: 1/1 L min^−1^ 121 kPa backpressure 86/86% RH	[113] Also in: [58,69,111,112]

^a^ Structures drawn based on information provided in respective sources. ^b^ Obtained via Mohr titration. ^c^ Obtained via back titration.

**Table 6:** Summary of AEI performances reported using PILs.

AEIs ^a^	Properties	Ref.
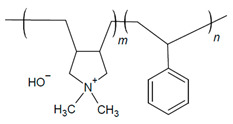 PolyDADMAC-*b*-Polystyrene	IEC ^b^: 1.03–3.4 PPD: 0.14 W cm^−2^ WU: 330% (3.4 IEC), 140% (1.03 IEC) AEMFC Conditions: 80 °C A/C Ionomer: PolyDADMAC-*b*-Polystyrene (IEC 1.03/IEC 3.40) A/C Catalyst (loading mg cm^−2^): PtRu (0.6 ± 0.07)/Pt (0.4 ± 0.03) Inlet A/C Fuel Conditions: 0.5/0.5 L min^−1^ No backpressure 100/100% RH	[118] Also in: [81]
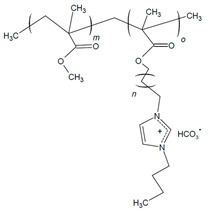 poly(MMA-b-MUBIm-HCO3)	IEC ^b^: 1.44 PPD: 0.0293 W cm^−2^ AEMFC Conditions: A/C Ionomer: poly(MMA-b-MUBIm-HCO3) A/C Catalyst (loading mg cm^−2^): PtRu (0.4)/Pt (0.4) Inlet A/C Fuel Conditions: 0.42 L min^−1^/1 L min^−1^ 100/100% RH 172 kPa backpressure	[114,117]
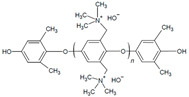 + 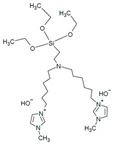 Quaternized poly(2,6-dimethyl-1,4-phenylene oxide) (QPPO) (top), ionic liquid (bottom) (QIL composite)	IEC ^c^: 3.3 (QIL-8) WU: 57–82% (at 80 °C) SR: 14.4–24.6% (at 80 °C) OH Conductivity: 135 mS cm^−1^ (at 90 °C, QIL-8) ORR Performance (Tafel): 58 mV dec^−1^ (QIL-8 with NIF-based catalyst)	[120]

^a^ Structures drawn based on information provided in respective sources. ^b^ Obtained via ^1^H NMR. ^c^ Obtained via back titration.

**Table 7:** Comparison of fabrication techniques for preparing electrodes.

Fabrication Method	Description	Aspects to Consider
CCM	Catalyst ink solution is applied directly to the membrane to create catalyst layer [127]	Less contact resistance; however, the AEM must have mechanical properties to withstand ink solution application [20,127]
CCS or GDE	Catalyst ink solution is applied to the GDL to create the catalyst layer [127]	Large active area and beneficial for low catalyst loading; however, prone to contact and transfer resistances [20,128]
Spray-coated	Spray-coating tool (e.g., airbrush, ultrasonic spray) is used to apply catalyst ink onto the AEM or GDL [117]	Need to ensure consistent flow for even coating Rapid solvent evaporation between coats [30] Helps form triple-phase boundary [117]
Hand-painted	Brush or applicator is used to coat the AEM or GDL [117]	Must wait a long time to dry between successive CL coats Requires even strokes to ensure uniform CL Helps form triple-phase boundary [117] Previously shown to be a better method with PIL AEIs [117]
Decal transfer	CL is coated onto a decal; once dried, the CL is then transferred from the decal to the AEM or GDL through methods such as hot pressing [117]	Heat pressing temperature and pressure can have negative impact on the MEA properties [117]
Screen printing	Utilize screen printing kits to deposit a catalyst ink paste to form the CL [131]	Consider solvent selection and its effects on viscosity and the AEM [131]
Blade coating	Utilizes a doctor blade apparatus that spreads a drop of ink solution on the material substrate until coating desired area [130]	AEM swelling if coating directly onto it [129] Can be used on a transfer decal and is more uniform than hand-painted [129]
Inkjet printing	Inkjet printer and cartridges used to deposit CL ink [121]	Can offer higher accuracy and create a graded ionomer content within the CL [121] Procedure includes extensive analysis during preparation steps to ensure ink is suitable for printer [121]

**Table 8:** Summary of performance results with Fumatech ionomer (AEI) FAA-3-SOLUT-10.

Solvent ^a^	AEI/Catalyst Ratio	A/C Catalyst (Loading mg cm^−2^)	AEMFC Performance (PPD W cm^−2^)	ECSA (m^2^ g^−1^)	AEM	Ref.
IPA + H_2_O	1:1	PtRu/Pt (0.4)	0.41	36.43	FAA-3-30 ^c,d^	[20]
	1.5:1		0.26 ^b^	27.38		
IPA + H_2_O	0.5:0.5	Pt/Pt (0.5)	0.067	69	FAA-3-PK-75 ^c,d^	[122]
	0.6:0.4		0.050	46		
IPA + H_2_O	1:1	PtRu/Pt (0.4)	0.84	35.95	FAA-3-30 ^c,e^	[20]
	1.5:1		0.48 ^b^	24.76		

^a^ Solvent ratio unspecified, ^b^ approximated based on figures presented in reference, ^c^ Fumatech GmbH, ^d^ CCS method, ^e^ CCM method.

**Table 9:** Summary and comparison of results with some ionomer changes for optimizing the CL.

AEI	Material Properties	Electrochemical Performance	CL Morphology Impacts	Ref.
CH_3_ Ionomer	IEC: 2.0 WU: ~50% ^−^OH Conductivity: 114 mS cm^−1^ (at 80 °C)	HFR: 14.9 mΩ PPD: 1.0 W cm^−2^ AEMFC Setup: 100% RH 0.1 MPa Backpressure PtRu/Pt (a/c catalyst) CCM	Oxygen stays on CH_3_ ionomer film (MD simulation) Less defined phase separation	[125]
CF_3_ Ionomer	IEC: 1.9 WU: ~30% ^−^OH Conductivity: 155 mS cm^−1^ (at 80 °C)	HFR: 11.6 mΩ PPD: 1.7 W cm^−2^ AEMFC Setup: 100% RH 0.1 MPa Backpressure PtRu/Pt (a/c catalyst) CCM	Oxygen diffuses into CF_3_ ionomer film (MD simulation) Defined phase separation	[125]
*meta*-QAPPT	IEC: 2.03 WU: 22.9 wt% ^−^OH Conductivity: 101.18 mS cm^−1^ (at 80 °C)	PPD: 0.995 W cm^−2^ AEMFC Setup: 95% RH 100 kPa backpressure PtRu/Pt (a/c catalyst) CCM	Dense, uniform Low water diffusivity (i.e., retain water for electrode hydration) Compact structure, strong interface with catalyst during RH changes	[23]
*para*-QAPPT	IEC: 2.05 WU: 35.91 wt% ^−^OH Conductivity: 112.70 mS cm^−1^ (at 80 °C)	PPD: 1.092 W cm^−2^ AEMFC Setup: 95% RH 100 kPa backpressure PtRu/Pt (a/c catalyst) CCM	Porous, aggregates High water diffusivity More likely to swell under dynamic conditions	[23]
*trans*-SB-DB	WU: 14.5% (at ~100%RH) Oxygen permeability: 4.62 (Barrer)	PPD: 0.64 W cm^−2^ AEMFC Setup: 100% RH No backpressure PtRu/Pt (a/c catalyst) CCS	ECSA: 59.9 m^2^ g^−1^ Agglomerated clusters with catalysts	[136]
*cis*-SB-DB	WU: 21.5% (at ~100%RH) Oxygen permeability: 7.98 (Barrer)	PPD: 1 W cm^−2^ AEMFC Setup: 100% RH No backpressure PtRu/Pt (a/c catalyst) CCS	ECSA: 92.4 m^2^ g^−1^ Maintain performance at low humidity (80%, 60%) Uniformly distributed catalyst, ionomer	[136]

## Data Availability

No new data were created or analyzed in this study. Data sharing is not applicable to this article.

## Abbreviations

The following abbreviations are used in this manuscript:

AEI	Anion exchange ionomer
AEM	Anion exchange membrane
AEMFC	Anion exchange membrane fuel cell
CL	Catalyst layer
DMSO	Dimethyl sulfoxide
ECSA	Electrochemical surface area or electrochemically active site
FLN	polyfluorene
GDE	Gas diffusion electrode
GDL	Gas diffusion layer
HER	Hydrogen evolution reaction
HOR	Hydrogen oxidation reaction
IEC	Ion exchange capacity
IL	Ionic liquid
IPA	Isopropanol
MEA	Membrane electrode assembly
MeOH	Methanol
ORR	Oxygen reduction reaction
PIL	Poly(ionic liquid)
PPD	Peak power density
QA	Quaternary ammonium
RH	Relative humidity
SR	Swelling ratio
WU	Water uptake
